# Whole-mount smFISH allows combining RNA and protein quantification at cellular and subcellular resolution

**DOI:** 10.1038/s41477-023-01442-9

**Published:** 2023-06-15

**Authors:** Lihua Zhao, Alejandro Fonseca, Anis Meschichi, Adrien Sicard, Stefanie Rosa

**Affiliations:** Department of Plant Biology, Swedish University of Agricultural Sciences (SLU), Uppsala, Sweden

**Keywords:** Cell biology, Biological techniques, Plant cell biology

## Abstract

Multicellular organisms result from complex developmental processes largely orchestrated through the quantitative spatiotemporal regulation of gene expression. Yet, obtaining absolute counts of messenger RNAs at a three-dimensional resolution remains challenging, especially in plants, owing to high levels of tissue autofluorescence that prevent the detection of diffraction-limited fluorescent spots. In situ hybridization methods based on amplification cycles have recently emerged, but they are laborious and often lead to quantification biases. In this article, we present a simple method based on single-molecule RNA fluorescence in situ hybridization to visualize and count the number of mRNA molecules in several intact plant tissues. In addition, with the use of fluorescent protein reporters, our method also enables simultaneous detection of mRNA and protein quantity, as well as subcellular distribution, in single cells. With this method, research in plants can now fully explore the benefits of the quantitative analysis of transcription and protein levels at cellular and subcellular resolution in plant tissues.

## Abstract (web summary)

Zhao et al. have developed a method to detect and quantify single molecules of RNA in several intact plant tissues. The method can also be used to simultaneously quantify and locate mRNAs and proteins in single cells with subcellular resolution.

## Main

Gene expression studies generally require a precise quantification of messenger RNAs of interest. These studies have commonly used bulk analysis, such as quantitative polymerase chain reaction with reverse transcription or RNA sequencing approaches. However, these methods do not provide information regarding cellular context and cell-to-cell variability in gene expression. Alternatively, a technique commonly used to study spatial patterns of gene expression is RNA in situ hybridization, but this technique is primarily qualitative. Furthermore, none of these techniques provides subcellular resolution. The development of single-molecule RNA fluorescence in situ hybridization (smFISH) has bridged this gap by allowing the detection of individual transcripts with subcellular resolution as well as precise quantification of the number of mRNAs in single cells^1,2^. The use of smFISH has revealed important insights into gene expression, including the presence of large cell-to-cell variability in mRNAs. smFISH has also enabled the measurement of specific gene transcription parameters, such as transcription and degradation rates, burst fractions and RNA half-life in single cells^3–7^.

In plants, smFISH was first applied to root meristem squashes of *Arabidopsis thaliana* (hereafter referred to as *Arabidopsis*)^8^. Plant tissues have very particular optical properties that are often challenging for the imaging process^9^. Thus, in plants, smFISH was initially applied to root squashes because this tissue has relatively low autofluorescence^8^. With this approach, loss of tissue morphology is, however, necessary to obtain monolayers of cells and thereby sufficiently reduce the fluorescence background to detect single molecules of RNA. Recently, smFISH has also been performed using paraffin embedding and sectioning protocols^10^. However, this procedure is lengthy and does not preserve the three dimensions of the tissues. Therefore, quantitative mRNA expression analysis at high resolution in simple preparations of intact plant tissues is still lacking. Although smFISH allows specific and quantitative analysis of gene transcription, it lacks information about the final gene products—proteins. Such information could, in principle, be acquired by combining mRNA detection with protein immunofluorescence but the existing protocols can be difficult to perform because they require sequentially hybridizing and imaging of mRNAs and proteins^11–14^. Also, protocols combining immunofluorescence with smFISH intact tissues have not yet been adapted to plants.

Here, we present a protocol for smFISH in plant whole-mount tissues enabling simultaneous detection of mRNA and proteins with cellular resolution in several intact tissues. To take full advantage of this protocol, we developed a computational workflow to quantify mRNA and protein levels at single-cell resolution. For this, we combined our mRNA and protein imaging with a cell wall stain to precisely assign molecular quantities to specific cells. To illustrate the potential of our method, we estimated the cellular specificity in gene expression using well-known protein reporter lines and determined the subcellular distribution of mRNAs known to be located in specific cellular compartments. With our smFISH whole-mount protocol and image analysis pipeline, we can now quantitatively analyse mRNAs and proteins at the cellular and subcellular levels in plants.

## Results

### smFISH for *Arabidopsis* whole-mount tissues

**Fig. 1:**
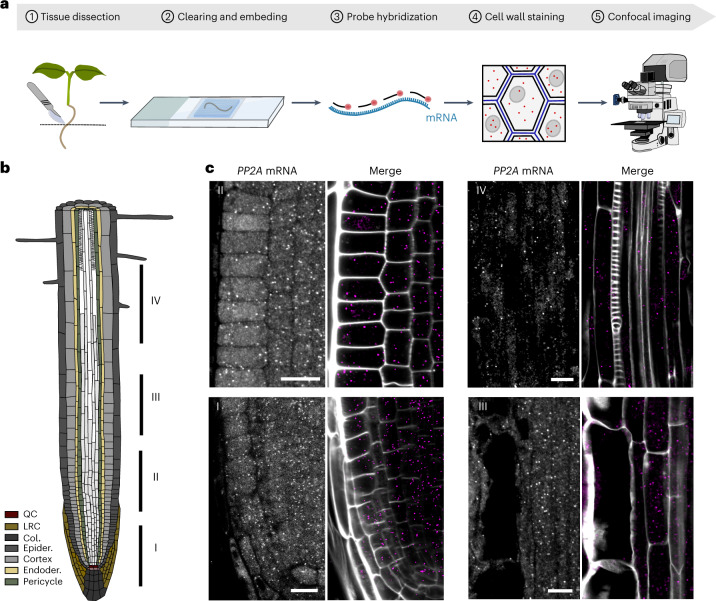
Single-molecule fluorescence in situ hybridization in *Arabidopsis* whole-mounts. **a**, Schematic diagram of the WM-smFISH method. **b**, Schematic representation of the different developmental regions (I–IV) in the *Arabidopsis* root. The different tissues including the quiescent centre (QC), lateral root cap (LRC), columella (Col.), epidermis (Epider.), cortex, endodermis (Enoder.) and pericycle are colour coded as indicated in the figure. **c**, Detection of *PP2A* mRNA molecules in *Arabidopsis* roots developmental regions I–IV. Left, *PP2A* smFISH channel (grey). Right, merged images with *PP2A* smFISH channel (magenta) and cell wall dye (white). The contours of cells were visualized through cell wall staining with Renaissance 2200. Scale bars, 10 μm. Experiments were repeated independently at least five times.

High levels of autofluorescence have prevented the detection of single RNA molecules in a broad range of plant tissues^8^. These difficulties are further complicated by the fact that smFISH is generally imaged using widefield optical microscopes, which are incompatible with the imaging of thick specimens. Assessing the three-dimensional distribution of RNA molecules implies preserving tissue integrity, optimizing the signal-to-noise ratio and preventing fluorescence from out-of-focus layers. The easiest way to overcome the latter is to use confocal microscopy, which allows the collection of optical sections of thick specimens. We thus tested whether the classical smFISH protocol would allow the detection of mRNA molecules in intact tissues using confocal imaging. To preserve the morphological integrity of the roots, we embedded the samples in a hydrogel according to the protocol published by Gordillo et al.^15^ (Fig. 1a). smFISH was performed using probes against the exonic regions of the *Protein Phosphatase 2A* (*PP2A*) mRNA and labelled with Quasar570 (Supplementary Table 1)^8^. *PP2A* is a housekeeping gene transcribed across a variety of tissue types and is relatively unaffected by abiotic and biotic stress^16^. Fluorescent spots from single mRNA molecules were visible but the signal-to-noise ratio was much lower than for squashed roots (Supplementary Fig. 1a,b). We, therefore, included additional clearing steps to further minimize autofluorescence and light scattering, including methanol and ClearSee treatments^17^, which substantially improved the signal-to-noise ratio (Fig. 1a and Supplementary Fig. 1b,c). Next, we added a cell wall staining step using Renaissance 2200 (ref. ^18^) to allow transcripts to be assigned to different cells and intracellular expression comparisons to be performed. In whole-mount root tips, *PP2A* mRNA signals could be observed as punctate dots evenly distributed through the cytoplasm (Fig. 1c). As expected, we were able to detect *PP2A* mRNAs across all cell types including the stem cell niche and differentiated cells within the root, without the need for cell wall digestion steps (Fig. 1b,c). We further confirmed that the signals observed correspond to true mRNA molecules by treatment with RNase A (Supplementary Fig. 1c,d,f). This shows that absolute mRNA counts can in principle be extracted with whole-mount smFISH (WM-smFISH) in connection with positional information and cell identities.

We then tested whether this method can be applied to other plant tissues, such as the shoot apical meristem (SAM), ovules and young leaves. With extended periods of ClearSee treatment (Supplementary Table 2), *PP2A* mRNA molecules can easily be detected in the SAM and ovule (Supplementary Fig. 2). However, in young leaves, *PP2A* transcripts were barely detected despite similar levels of background fluorescence, probably because there was very low or no expression present in this tissue. Therefore, we decided to test another housekeeping gene generally expressed at higher levels. *NAD-dependent glyceraldehyde-3-phosphate dehydrogenase* (*GAPDH*) is a gene that encodes for a ubiquitous enzyme having essential roles in plant metabolism often used as a reference gene^16^. We have designed probes against the mRNA of *GAPDH* isoform *GAPC2* (At1g13440) and labelled them with Quasar670, which emits at wavelengths in which autofluorescence levels are lower (Supplementary Table 1 and Supplementary Fig. 3). Transcripts of this gene were clearly detected in young leaves (Supplementary Fig. 4), as well as in other tissues including embryos, anthers, petals and carpels (Supplementary Fig. 5). To determine whether WM-smFISH could be applied broadly, we next tested the ability of this method to detect *GAPDH* transcripts in a monocot species, *Hordeum vulgare* (barley) (Supplementary Fig. 6 and Supplementary Table 1). Transcripts could be easily detected in both differentiated leaves and inflorescence tissues, suggesting that smFISH can be easily applied across angiosperm species. These results demonstrate that single molecules of endogenous transcripts can be detected on a broad range of whole-mount plant tissues with different fluorophores and high specificity and resolution.

### Single-cell detection and quantification of mRNA and protein

**Fig. 2:**
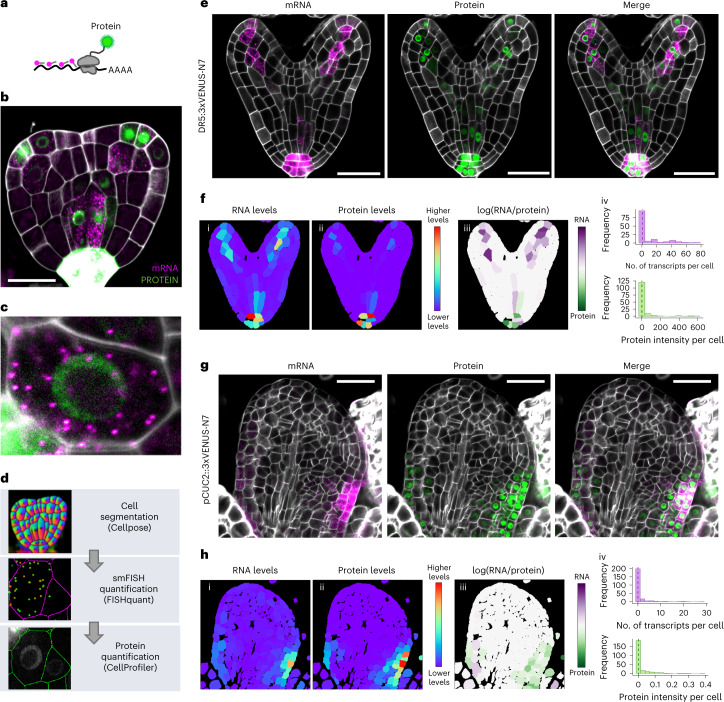
WM-smFISH enables combining of RNA and protein quantification. **a**, Schematic diagram for simultaneous RNA and protein detection. *VENUS* mRNAs are hybridized and detected with smFISH probes and the VENUS proteins are detected directly through protein fluorescence. **b**, Transition embryo expressing *pDR5rev::3xVENUS-N7* showing detection of *VENUS* mRNA (magenta) and VENUS protein (green). **c**, Close-up of a single cell from the embryo presented in **b**, showing individual mRNAs as single spots and VENUS protein fluorescence in the nucleus. **d**, Workflow diagram showing the three-stepped pipeline for quantitative analysis of WM-smFISH with fluorescent protein detection. **e**–**h**, Simultaneous mRNA and protein detection in heart stage embryo (**e**) and leaf (**g**) using *pDR5rev::3xVENUS-N7* and *pCUC2::3xVENUS-N7* reporter lines, respectively. Confocal microscopy images for mRNA (magenta), protein (green), and merged signals. Quantification results for mRNA and protein in heart stage embryo (**f**) and leaf (**h**). (i, ii) Heatmaps represent the levels of the mean signal intensity per cell detected in each channel (for RNA or protein detection). (iii) Heatmap representing the ratio between the RNA and protein signal intensities per cell. (iv) Histograms showing the distribution of the number of transcripts (magenta) or total protein intensity (green) per cell, the median value is indicated with a dashed line. The contours of cells were visualized with Renaissance 2200 dye (white). Scale bars, 20μm. Experiments were repeated independently at least three times.

Although smFISH can provide precise and quantitative measurements of gene expression, it lacks information at the protein level. To that end, we thought to combine WM-smFISH with the detection of fluorescent reporter proteins. We designed probes that targeted the mRNA of VENUS fluorescent protein (Supplementary Table 1), with the aim to simultaneously detect the protein and transcripts expressed by the same transgene (Fig. 2a). As a proof-of-concept, we analysed the auxin signalling reporter line *pDR5rev::3xVENUS-N7* and a reporter line for the NAC transcription factor *CUP-SHAPED COTYLEDON* 2, *pCUC2::3xVENUS-N7*, both of which have been extensively characterized^19,20^ (Fig. 2b,e,g). We chose to perform this analysis on reporter constructs containing three concatenated fluorescent reporters to improve the signal and facilitate the detection of mRNAs in green tissues, such as leaves and the inflorescence meristem. Indeed, 90 fluorescent probes can bind to the mRNAs of these transgenes (*3xVENUS*) as opposed to 48 for the *PP2A* transcripts. We first examined the detection of mRNA and protein in whole-mount *Arabidopsis* young leaves, floral primordia, ovule, embryos and roots (Fig. 2b,e,g and Supplementary Fig. 7). The signal-to-noise ratio improved substantially, and mRNA dots could be easily visualized in leaves and inflorescence tissues (Fig. 2b,c,e,g and Supplementary Fig. 7). Importantly, the fluorescence of the reporter is well-preserved throughout the WM-smFISH procedure allowing cellular comparison of mRNA and protein distribution (Fig. 2b,c). We have also tested whether ClearSee treatment affected reporter fluorescence and RNA detection (Supplementary Fig. 8). Consistent with previous observations on fluorescent reporter signal^17^, our results showed that ClearSee improved both protein and RNA signals, further indicating that it is important to compare samples that have been treated similarly.

To appreciate the spatial differences in the distribution of mRNAs within tissues, we developed a computational workflow to quantify mRNA dots with cellular resolution using WM-smFISH images (Fig. 2d). In brief, it segments two-dimensional confocal images based on cell wall (SR2200 dye) signal using Cellpose^21^ then uses these cell outlines to estimate the number of mRNA foci per cell using FISH-quant^22,23^ and measures the protein intensity fluorescence with CellProfiler^24^. A colour scale reflecting intensities (for protein and RNA levels) was finally used to label the segmented cells throughout the confocal images (Fig 2f(i,ii),h(i,ii)). To visualize the variation in the ratio between mRNA molecules and protein accumulation, we generated heatmaps with the log ratio between the intensity of WM-smFISH and VENUS fluorescent signals (Fig. 2f(iii),h(iii)). Here, we choose to use fluorescence intensity rather than the number of mRNA molecules to compare similar measurements. To do this, we first verified that the fluorescence levels per cell correlate with the number of transcripts (Supplementary Fig. 9a,b). The resulting distribution heatmap allows a quantitative and spatial visualization of expression and protein distribution patterns. Histograms can also be used to plot the number of transcripts and protein levels per cell in multiple samples (Fig. 2f(iv),h(iv)).

To validate our quantification workflow, we first measured the number of *PP2A* mRNA molecules in root samples treated with RNase. As expected, in RNase-treated samples, the majority of cells did not show any *PP2A* transcripts, confirming that this pipeline specifically quantified mRNA foci (Supplementary Fig. 1c,e,f). Also, our automated detection and counting gave a similar distribution of transcripts per cell as the manual counting of mRNA dots in squashed roots (Supplementary Fig. 10f). Next, we asked whether different image acquisition modes could affect the detection of mRNA dots. We obtained similar distributions for the number of transcripts per cell with widefield and confocal microscopes (Supplementary Fig. 10g). We, nevertheless, observed significant differences in automated counting of transcripts between root squashes and whole-mount roots. The distribution of mRNA counts in squashed roots was slightly shifted toward lower values, probably because of differences in the population of cell types analysed by both methods (Supplementary Fig. 10h). Indeed, not all cell types are evenly isolated when squashing the roots, which may tend to bias the proportion of cells analysed. However, the values obtained with both methods are within the same range of mRNA molecules per cell, indicating that WM-smFISH does not compromise transcript detection. Also, quantification of the number of transcripts per cell in different z-slices within a root and between roots confirmed the accuracy and consistency of mRNA detection in deep tissues (Supplementary Fig. 11) and among replicates (Supplementary Fig. 12). Furthermore, we estimated the cellular specificity of our quantification pipeline by correlating mRNA counts with the level of the corresponding protein in the *pDR5rev::3xVENUS-N7* line (Supplementary Fig. 13). The VENUS protein levels and mRNA counts were significantly correlated (Pearson *R*^2^ = 0.3955) (Supplementary Fig. 13c), contrasting with the lack of correlation between the number of *PP2A* transcripts and VENUS protein intensity per cell (Pearson *R*^2^ = 0.0354) (Supplementary Fig. 13d). The pipeline is also applicable for the detection of endogenous transcripts in both plant species analysed (Supplementary Figs. 2b,c, 4 and 6b–d). Despite the low numbers of *PP2A* transcripts per cell in the SAM and ovule (Median_SAM_ = 1; Median_ovule_ = 2; Supplementary Fig. 2b,c) this workflow worked well, confirming that even low levels of cellular expression can be quantified by WM-smFISH. In addition, this confirmed that, contrary to *GAPDH*, *PP2A* is either extremely low or not expressed in young leaves. In barley leaves, the software could also accurately distinguish between mRNA molecules from the remaining autofluorescence surrounding the vascular bundle (Supplementary Fig. 6). Moreover, on average only 0.205 ± 0.629 mRNA molecules per cell were detected after RNase treatment across the tissues tested, despite the presence of large autofluorescent structures in some tissues (Supplementary Fig. 6d). Such a low detection error will not affect the quantification of most transcripts but could become non-negligible for transcripts with extremely low expression levels. In such cases, control RNase treatment should be systematically considered. Overall, these results validate the accuracy and specificity of our quantification method and indicate that this automated workflow is a useful tool for quantifying mRNA levels in whole-mount tissues. Moreover, because this approach can be combined with the visualization of fluorescent reporter proteins, it also has the potential to be used to model transcription/translation dynamics, assess intercellular protein or RNA movement and analyse colocalization between mRNA and proteins.

We further applied our quantification approach to investigate expression of the *VENUS* reporters at the protein and mRNA levels in different tissues (Fig. 2f,h and Supplementary Figs. 13a and 14). Globally, the spatial distributions of the mRNA molecules and protein signals throughout the tissues were in good agreement with each other and followed the known expression pattern for the two reporter constructs^19,20,25,26^. We nevertheless did not observe a full expression overlap between the mRNA and protein signals in all tissues. For instance, in the embryo, we observed several cells with high mRNA to protein ratios; this often occurs in cells that express low levels of mRNA (Fig. 2f and Supplementary Fig. 9c). RNA detection by WM-smFISH may therefore be more sensitive than reporter protein imaging. One possible interpretation is that, at this developmental stage, the auxin response has been newly activated in these cells such that the reporter proteins have not yet been translated. Similar discrepancies were also observed in leaf and inflorescence tissues (Fig. 2g,h and Supplementary Figs. 9d and 14). For instance, in the young leaf of the *pCUC2::3xVENUS-N7* line, the reporter proteins are more broadly distributed than the mRNA molecules (Fig. 2h). *pCUC2::3xVENUS-N7* mRNAs appear in cells along leaf margins, which seems more consistent with the well-established CUC2 function in leaf serration patterns^27^. The diffusion of fluorescent proteins to the neighbouring cells seems unlikely owing to the high molecular mass of the three concatenated fluorescent proteins and the presence of nuclear localization signals^28^. Therefore, these differences are probably linked to the reporter proteins’ stability considerably exceeding mRNA stability. In this way, the protein signal could persist within a cell even when transcription is not taking place. In dividing tissues such as young leaves and inflorescence meristem, reporter protein distribution could further be extended through cell division, although mRNA molecules would mostly remain in transcriptionally active cells. These results illustrate that fluorescent reporters’ imaging can be combined with WM-smFISH to provide quantitative information on gene activity, with the latter delivering a closer view of the spatial distribution of gene transcription.

### Quantification of cellular response to exogenous stimuli

**Fig. 3:**
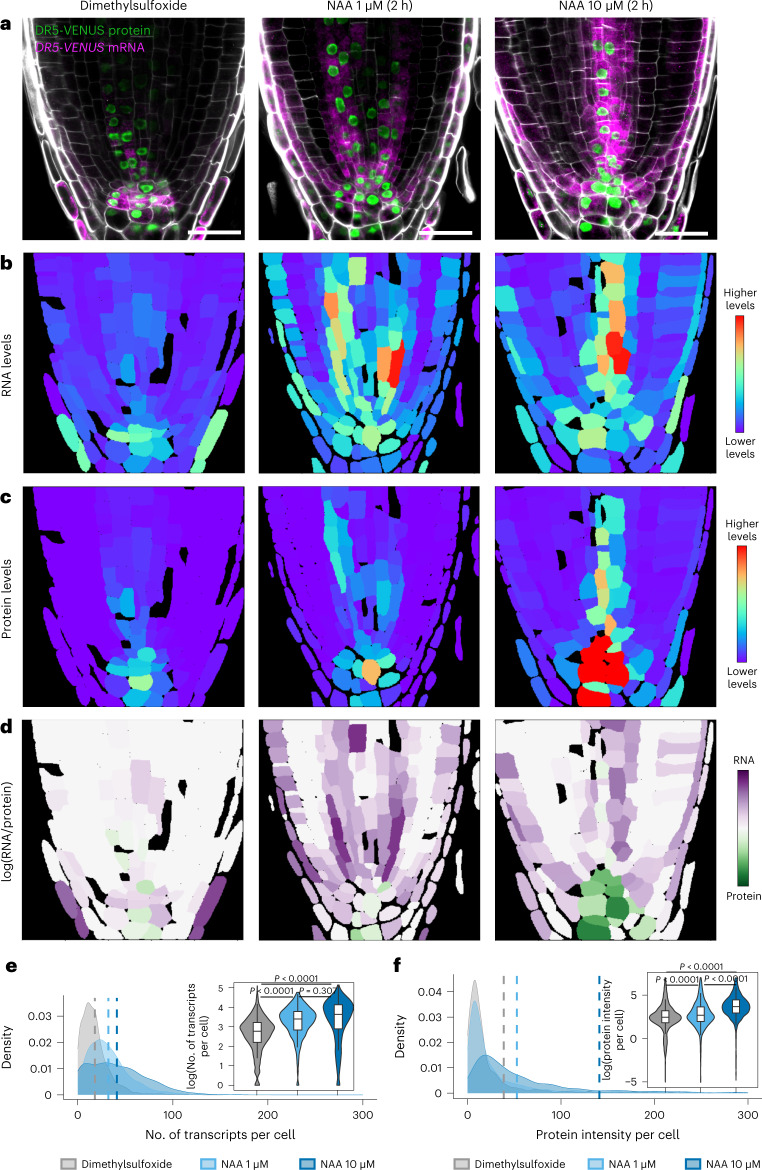
WM-smFISH enables spatial and quantitative characterization of gene expression at RNA and protein levels upon exogenous stimulus. **a**, Representative images for the detection of VENUS mRNA (magenta) and protein (green) in *pDR5rev::3xVENUS-N7* reporter seedlings treated with dimethylsulfoxide, NAA 1 µM, or NAA 10 µM for 2 h. The contours of cells were visualized with Renaissance 2200 dye (white). Scale bars, 20 μm. **b**,**c**, Heatmaps representing the levels of the mean signal intensity per cell detected in the channels for RNA (**b**) or protein (**c**) detection in the representative images shown in **a**. **d**, Heatmaps representing the ratio between the RNA and protein signal intensities per cell in the representative images shown in **a**. **e**,**f**, Density plots showing the distributions for the number of transcripts (**e**) and total protein intensity (**f**) per cell detected in all the treated roots (*n* for dimethylsulfoxide = 1,659 cells examined over 10 roots, *n* for NAA 1 µM = 1,830 cells examined over 10 roots, *n* for NAA 10 µM = 1,456 cells examined over 9 roots). The dashed lines represent the mean values for each condition. Violin plots showing the log-normalized (base *e*) distributions. Boxes inside show the interquartile range (IQR; 25%–75%), indicating the median values as a horizontal line. Whiskers show the ±1.58× IQR value. The *P* values for analysis of variance followed by one-sided Tukey’s HSD tests are shown in the inserts: dimethylsulfoxide versus NAA 1 µM (*P* < 0.0001), dimethylsulfoxide versus NAA 10 µM (*P* < 0.001), NAA 1 µM versus NAA 10 µM (*P* = 0.307) (**e**); dimethylsulfoxide versus NAA 1 µM (*P* < 0.0001), dimethylsulfoxide versus NAA 10 µM (*P* < 0.001), NAA 1 µM versus NAA 10 µM (*P* < 0.0001) (**f**). Experiments were repeated independently two times.

We further tested our method by analysing the expression profile of *pDR5rev::3xVENUS-N7* in *Arabidopsis* roots in response to the exogenous application of the synthetic auxin naphthalene-1-acetic acid (NAA). In this experiment, we used two different concentrations (1 and 10 µM) to evaluate the difference in sensitivity between WM-smFISH and fluorescent reporter imaging and determine whether we could measure quantitative differences in transcript accumulation. A dose-dependent induction in RNA and protein levels was observed (Fig. 3a–c,e,f). Globally, we observed a coordinated increase in protein and mRNA in the quiescent centre and stele cells. However, mRNA signals increased in the epidermis cells without any apparent activation of the reporter protein fluorescence. The quantification of mRNA levels or protein fluorescence intensity per cell further confirms a higher increase in mRNA compared with protein at lower NAA concentrations (Fig. 3b–f). These results are, therefore, consistent with WM-smFISH being more sensitive. They also demonstrate that combining WM-smFISH with reporter protein imaging can provide quantitative spatiotemporal information on the transcriptional–translational dynamics of gene expression. Combining these measurements with positional information and three-dimensional cell atlas^29–32^ could provide powerful tools to assess the influence of cellular context on gene expression and translation at a fine scale.

### Subcellular detection and colocalization of mRNA and protein

**Fig. 4:**
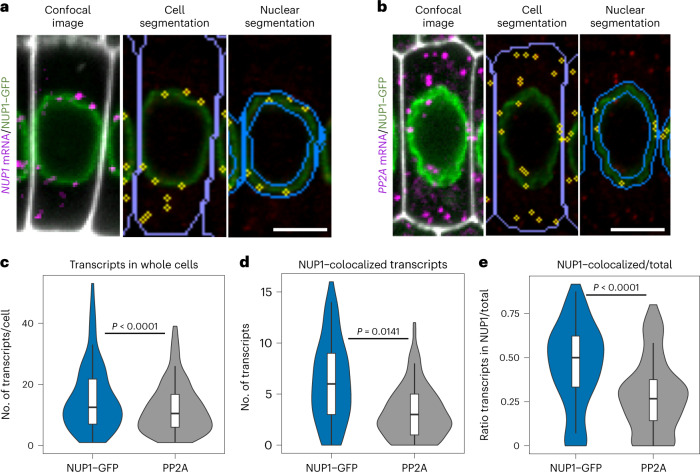
RNA detection by smFISH can be combined with protein detection for subcellular colocalization analysis. **a**,**b**, Representative images to evaluate the subcellular localization of *NUP1–GFP* (**a**) or *PP2A* mRNAs (**b**) in cells from the meristematic zone in *NUP1–GFP*-expressing roots. Confocal images show the simultaneous detection of the respective mRNA (magenta), NUP1–GFP protein (green) and contours of cells from the Renaissance 2200 dye (white) (left). Cells and nuclear envelope were segmented based on Renaissance 2200 and NUP1–GFP signals, respectively. The detected mRNA molecules are highlighted in yellow either in the whole cell (middle) or colocalizing with the NUP1–GFP signal (right). Scale bars, 5 μm. **c**–**e**, Violin plots showing the number of *NUP1–GFP* or *PP2A* mRNA molecules per cell (*n* for NUP1–GFP = 97 cells examined over seven roots, *n* for PP2A = 141 cells examined over seven roots). Boxes show the IQR (25%-75%), indicating the median values as a horizontal line. Whiskers show the ±1.58× IQR value. A *t* test was performed to compare both mRNAs, the *P* value is indicated on the graph: *P* < 0.0001 (**c**), *P* = 0.0141 (**d**), *P* < 0.0001 (**e**). The plots show the number of transcripts per cell (**c**), the number of transcripts colocalizing with the NUP1–GFP protein (**d**) and the ratio between the number of colocalized transcripts and the total number of transcripts per cell (**e**). Experiments were repeated independently two times.

The subcellular localization of RNAs is important to regulate biological processes, allowing them to find their target, control their translation or regulate their stability^33,34^. One example is the mRNAs of nucleoporins (NUP1/NUP2), which are localized and translated next to the nuclear envelope to ensure proper delivery of the proteins to the nuclear pore complex in yeast^35^. We tested whether WM-smFISH can be used to quantitatively evaluate mRNA subcellular localization patterns by colocalizing mRNA spots with fluorescent protein signals. For this, we adapted our automated workflow to segment the protein signal and quantify the number of mRNAs colocalizing with the reporter protein. Using our workflow, we examined the subcellular distribution of *NUP1* mRNA in the apical meristem of *Arabidopsis* roots expressing NUP1–GFP (green fluorescent protein) (Fig. 4a). We used *VENUS* probes (Supplementary Table 1) to detect the *GFP* mRNA and assess the mRNA position, which we compared with the localization of the nuclear envelope using the NUP1–GFP signal (Fig. 4a). As a control, we performed WM-smFISH using *PP2A* probes, which we have previously shown to be evenly distributed throughout the cytoplasm^8^ (Fig. 4b). We detected a significantly higher (*P* < 0.0001, Student’s *t* test) number of *NUP1* transcripts colocalizing with NUP1–GFP protein compared with *PP2A* (Fig. 4d). Nevertheless, we also observed a slightly higher number of *NUP1–GFP* transcripts per cell compared with *PP2A* (*P* < 0.0141, Student’s *t* test) (Fig. 4c). We therefore normalized the number of transcripts colocalizing with NUP1–GFP signal by the total number of mRNAs per cell to ensure that a higher proportion of transcripts colocalized with NUP1–GFP. On average, 45.4% of the *NUP1* mRNAs colocalized with NUP1–GFP, whereas only 28.7% of *PP2A* transcripts are present within the nuclear envelope (Fig. 4e). The differences (*P* < 0.0001, Student’s *t* test) indicate that *NUP1* mRNA is preferentially targeted to the nuclear envelope. This illustrates how WM-smFISH can be used in combination with different fluorescent reporters to explore mRNA subcellular localization or to visualize their colocalization with protein partners. Although technically more challenging, we also demonstrate that WM-smFISH and immunofluorescence imaging can be combined in a sequential manner (Supplementary Fig. 15), allowing for colocalization studies with endogenous proteins, even in species for which transgenesis has not yet been established.

## Discussion

In conclusion, we have developed a method that allows the detection of single RNA molecules in a wide range of plant organs and species. WM-smFISH can be combined with fluorescent protein reporters or immunofluorescence imaging to provide simultaneous detection and quantification of mRNAs and proteins with cellular and subcellular resolution. Our automated quantification workflow was able to detect a broad range of cellular expression levels (from 1 to >50 mRNA molecules per cell). This method is rapid, can be easily implemented and allows absolute counts of mRNA molecules in whole-mount tissues. However, because WM-smFISH requires at least 30 probes of 18–20 nucleotides each, it does not work for RNAs shorter than 600 nucleotides. Also, for tissues with high autofluorescence, it is important to carry out long clearing treatments otherwise background autofluorescence may mask RNA signals, resulting in detection failure or false-positives in the case of a punctuated background. The latter issue will not affect the quantification of most genes, but could become significant for genes with extremely low expression levels. The signal-to-noise ratio can also be improved with the selection of appropriate fluorophores, with emission in wavelengths where tissue autofluorescence is lower. We used two different fluorescent dyes, Quasar570 and Quasar670; because autofluorescence seemed generally lower in the far-red range, the choice of this fluorophore may be preferable for plant tissues with high autofluorescence. However, plant tissues contain multiple autofluorescent compounds, and the choice of fluorophore may need to be adjusted accordingly. For instance, Quasar570 has been reported to be brighter and more photostable (Stellaris, Biosearch Technologies) and works well in tissues with low autofluorescence, such as roots (see Supplementary Table 3 for additional strengths and limitations of WM-smFISHs).

Determining when and in which tissues and cell types a gene is expressed is essential for their functional characterization. WM-smFISH will be an important tool to model the transcription/translation dynamics and investigate regulatory mechanisms associated with developmental and physiological processes, providing exciting new opportunities for plant research.

## Methods

### Plant materials

pDR5rev::3xVENUS-N7 (N799364) and pCUC2::3xVENUS-N7 (N23896) lines were obtained from the Eurasian Arabidopsis Stock Center (uNASC). The NUP1-GFP seeds were a gift from C. Liu. Barley (*Hordeum vulgare*) seeds were a gift from S. Moreno (Department of Plant Biology, SLU – Uppsala, Sweden). All plants were grown as described in the Supplementary Methods.

### Sample preparation

Paraformaldehyde-fixed samples were permeabilized and cleared through a series of methanol, ethanol and ClearSee^17^ treatments before being embedded in an acrylamide polymer in which the hybridization was performed. The Supplementary Methods give additional details on the sample preparation and embedding steps.

### In situ hybridization

SmFISH probe design and hybridization conditions for different *A. thaliana* and *H. vulgare* tissues are described in the Supplementary Methods.

### Imaging

Whole-mount and squashed plant tissues were imaged with a Zeiss LSM800 confocal microscope as described in the Supplementary Methods.

### Image processing and analysis

Cell segmentations were performed using Cellpose^21^. RNA foci were detected and counted using FISH-quant-v3 (ref. ^23^). Colocalization analysis and heatmap reconstruction were performed using CellProfiler^24^. Additional details on the image processing and analyses can be found in the Supplementary Methods.

### Combined smFISH with immunofluorescence

To combine mRNA and protein detection by immunofluorescence in whole-mount, we established a sequential WM-smFISH–immunofluorescence protocol. mRNA signals were first imaged following the WM-smFISH as described in the Supplementary Methods. Subsequently immunostaining of Histone H4 proteins was carried out using the protocol described in Rosa et al.^36^.

### Reporting summary

Further information on research design is available in the Nature Portfolio Reporting Summary linked to this article.

## Supplementary information

Supplementary Information Supplementary Figs. 1–15, Tables 1–3 and Methods.

Reporting Summary

## Data Availability

All the raw microscopy images used in this manuscript are openly available in Figshare at 10.6084/m9.figshare.22699132.
