# Supplementary material for: Whole-mount smFISH allows combining RNA and protein quantification at cellular and subcellular resolution
Source: Nat Plants. 2023 Jun 15;9(7):1094–102. doi: 10.1038/s41477-023-01442-9 (PMC10356603; doi:10.1038/s41477-023-01442-9)
Supplement: Supplementary file 1 — Supplementary Figs. 1–15, Tables 1–3 and Methods. [file 41477_2023_1442_MOESM1_ESM.pdf]

nature plants

Article

https://doi.org/10.1038/s41477-023-01442-9

Whole-mount smFISH allows combining RNA and protein quantification at cellular and subcellular resolution

In the format provided by the
authors and unedited

Supplementary Methods

Plant growth condition

Arabidopsis seeds were surface sterilized in 5% (v/v) sodium hypochlorite for 5 min and rinsed three times in sterile distilled water. Seeds were then stratified for 2 days at 4°C before germination in a growth chamber in a vertically oriented Petri dish containing Murashige and Skoog (MS) media (Gamborg et al. 1976). Plants were grown under a photoperiod of 16 hours day and 8 hours night and a temperature cycle of 22°C during the day and 20°C during the night. Seven-day-old seedlings were transferred to soil and grown at 22°C under long-day conditions (16 hours day and 8 hours night) in a phytotron and grown for tissue harvesting.

In this study, root tips (approximately 1 cm) were collected 7 days after germination. Young leaves were dissected from 10-day old seedlings. The embryos were isolated from 3 to 5-day old seeds. Floral tissues were collected 5-6 weeks after germination. The auxin treatment was performed on plates with 7-day old seedlings using 1 or 10 µM NAA (Sigma-Aldrich, Cat.# N0640) for 2 hours.

Barley (*Hordeum vulgare*) seeds (variety Golden promise) were surface sterilized in 5% (v/v) sodium hypochlorite for 10 min and rinsed three times in sterile distilled water. Seeds were then germinated in Petri dishes containing Murashige and Skoog (MS) media. Seedlings were grown under the same light and temperature conditions described above for Arabidopsis. After 7 days of growth, samples were dissected by manually cutting the basal part of young leaves. Barley inflorescence tissues were dissected from about one-month-old plants.

Probe design and preparation

Probes directed against the genes of interest (*PP2A* and *Venus*) were designed using the LGC Biosearch Technologies' Stellaris® version 4.2. The sequences of the probe used in this study are shown in Table S1 and S2. The probes were diluted in TE buffer at a final stock concentration of 25 µM.

smFISH procedure on root squashes

Root tips of 7-day-old seedlings were cut and fixed in 4% paraformaldehyde (In PBS buffer, pH 8.0, Sigma-Aldrich, Cat.#6148). The roots were then washed and squashed manually between the slide and coverslip. For a detailed description of smFISH on root squashes see Duncan *et al*. (Duncan et al. 2016).

Whole-mount smFISH

I. Fixation, preparation and clearing of Arabidopsis roots

1. Place the dissected samples into a small glass dish (Watch Glass, Square, 1 5/8 in, Carolina, Cat.#742300) containing freshly prepared 4% paraformaldehyde.
2. Incubate for 30 min at room temperature under a fume hood.
3. Wash two times with 1x PBS (PH 7.4, Thermo Scientific, Cat.# AM9624).

4. Immerge the samples in 100% Methanol for 15min twice.
5. Incube with Ethanol 100% twice for 15min.
4. Clear the tissues in ClearSee at 4°C in the dark. The times of incubation depend on the tissues and are given in Table S3.
5. Wash two times with Stellaris wash buffer A (Stellaris, Cat.#SMF-WA1-60) for 15 min.

II. Embedding of Arabidopsis roots

1. Arrange the samples onto a poly-L slide (Thermo Scientific, Cat.#10219280) and remove as much wash buffer as possible without letting the sample dry before embedding.
2. Add activated embedding solution (20µl to each slide) and cover with a coverslip. Note: The addition of tetramethylethylenediamine (TEMED) in the embedding solution activates the polymerization. This should only be done just before step 2 when the samples have been arranged on the slide.
3. Let the polymerization occurs at room temperature for at least 20 min.
4. Carefully remove the coverslip.

III. Probe hybridization

1. To prepare a hybridization solution, add 0.5µl of each required probe set stock solution to 100 µl of hybridization buffer (10% dextran sulfate, 2 × SSC and 10% formamide) to reach a final concentration of 250 nM.
2. Add 100 µl of the hybridization solution to each slide, cover with a coverslip to prevent evaporation and incubate in a humid chamber at 37 °C overnight in the dark.

IV. Sample mounting

1. Remove the coverslip and place the slides in an opaque coplin jar.
2. Submerge the slides two times in 50ml of the wash buffer B for 30 min.
3. Remove the slides from the coplin jar.
4. Stain the samples at 37 °C for 10 min in the dark by adding 100 µl of the desired dye solutions (DAPI or cell wall dye) to each slide. Cover the sample with a coverslip. In the study, we used the SCRI Renaissance 2200 solution (see Recipes, (Musielak et al. 2015)) to mark the cell walls.
5. Remove the coverslip, place back all the slides in the opaque coplin jar and immerse the slides in 50ml of wash buffer B for 15min.
6. Remove the slide from the staining dish and eliminate the excess of the buffer.
7. Mount each slide with 20 µl of vectashield (BioNordika, Cat.# H-1000-10).
5. Cover the samples with a coverslip, remove excess vectashield and seal with nail varnish.
6. Image the slides the same day to avoid fluorophore fading.

V. Imaging

Samples were imaged on a Zeiss LSM800 inverted confocal microscope (Zen Black Software) using a 63X water-immersion objective (1.20 NA). A series of optical sections with z-steps of 0.22 µm were collected. Images were captured with a 561 nm excitation line and emission was collected at 535 - 700 nm for probes

labeled with Quasar570. For imaging VENUS protein we used a 515 nm excitation line and emission was collected at 400 - 550 nm. For GFP, an excitation line of 488 nm was used and emission at 410 - 540 nm. For SCRI Renaissance 2200 imaging we used a 405 nm laser line and and emission was collected at 400 - 600 nm. For widefield microscopy, we used a cooled quad-port CCD (charge-coupled device) ZEISS Axiocam 503 mono camera. The following wavelengths were used for fluorescence detection: for probes labeled with Quasar570, an excitation filter of 533-558 nm was used with signal detection at 570-640 nm; for SCRI Renaissance 2200, an excitation filter of 335-383 nm with signal detection at 420–470 nm.

VI. Image processing and analyses

Images were analyzed using a computational workflow based on three freely available software: Cellpose (Stringer et al. 2021), FISH-quant (Mueller et al. 2013, Imbert et al. 2022); and CellProfiler(Stirling et al. 2021). All these programs can be either used from the command line or through graphical interfaces. We used the versions with graphical interfaces in this protocol to generate a more user-friendly method. For the different analyses, we worked with separated TIFF images for each channel (SR2200, smFISH, and GFP), which were obtained from the raw CZI files.

For cell segmentation, the SR2200 signal (membrane marker) was inverted using Cellprofiler, and then utilized as input to generate cell mask files with Cellpose. We run Cellpose with an interface using the plugin “SegmentObjects” (object type: Cells) in ImJoy (Ouyang et al. 2019). This plugin and a manual for users are freely provided by Florian Mueller on Github (https://github.com/fish-quant/fq-segmentation).

To detect the RNA foci in the red- or far-red-channel, we used the Matlab version of the software FISH-quant v3(Mueller et al. 2013). The software and manual are provided on Bitbucket by Florian Mueller (https://bitbucket.org/muellerflorian/fish_quant/src/master/). The cell mask images obtained from Cellpose were used to write a text file with the cell outline coordinates employing the “Cell segmentation” tool in FISH-quant. For each experiment, one image was analyzed to define the pre-detection settings for background subtraction and spots Gaussian fluorescence fitting control based on the point-spread function (PSF). These settings were used to run the analysis in batch mode to obtain the preliminary results. False positives were removed by thresholding the Sigma-XY, amplitude, and pixel-intensity parameters, following the developer’s advice. The coordinates and number of transcripts per cell were exported as tabulated text files, keeping an identifier for each segmented cell from each image file analyzed.

We designed a Cellprofiler pipeline to analyze the protein levels from the GFP channel. In order to obtain the protein information at the single-cell level, we imported the cell mask file (obtained with Cellpose), allowing us to keep the same identifiers with which the RNA quantification was performed. Then, the integrated and mean intensity of protein fluorescence could be quantified and exported as CSV files.

To perform the colocalization analyses we imported the FISHquant detection results into Cellprofiler. To this end, the dot coordinates text files (obtained from FISHquant) were used to create binary mask TIFF images with ImageJ macros, indicating the localization of the dots as regions of interest (ROIs). The nuclear envelope was outlined using the GFP signal from NUP1-GFP. The number of transcripts within the outlined regions was counted and compared with the total number of transcripts per cell.

For graphical representations of the protein and RNA levels, heatmap images were generated using Cellprofiler. For this, cell outlines and RNA dot coordinates were imported as explained above, and CY3 and GFP signal mean intensity per cell were calculated. These values were used to represent the protein and RNA levels per cell with a color scale. Furthermore, we calculated the logarithmic ratio of CY3 and GFP intensity per cell to the accumulation of protein and RNA molecules. These values were also represented as heatmaps, as described above.

All the presented Cellprofiler pipelines are freely available on the Cellprofiler website (https://cellprofiler.org/published-pipelines). The density, violin, scatter plots, and statistical analyses presented in this work were performed using ggplot2 or base R-packages.

Combined smFISH with Immunofluorescence

We performed a sequential smFISH-immunofluorescence by first performing the RNA FISH as describe above before performing immunostaining using the protocol described in Rosa et al., 2016. Briefly, after imaging RNA by smFISH slides were washed with 1X PBS (three times for 10min) and then digested in a mixture of 1% driselase, 0,5% cellulase, 1% pectolyase) at 37℃ for 30 min. Roots were then washed three times in 1X PBS for five minutes each and blocked with 4% BSA in PBS for 50 min. The sample was then incubated with a rabbit anti-Histone H4 antibody (AHP418, Bio-rad; in 1% BSA, 1X PBS) at 1:100 dilution. The slides were incubated at 4°C for overnight in a humid box. After washing the slides in 1X PBS for 5 min each, samples were incubated with a secondary antibody (goat Alexa Fluor 488 anti-rabbit; Invitrogen, A-11001) at a 1:200 dilution for 1 h in a humid dark box at 37℃. After washing the slides three times in 1X PBS for 5 min each, samples were mounted in Vectashield. In order to find the cells previously imaged for smFISH, we saved the stage positions of those cells at the microscope.

Media Recipes:

smFISH probe stock:
Probe sets were resuspended in Tris-EDTA (PH 8.0, sigma-Aldrich, Cat.#93283-500ML) to a final concentration of 25 µM.

Clearsee solution (adapted from (Kurihara et al. 2015)):
-Xylitol powder [Sigma-Aldrich, Cat.#X3375-100g, final 10% (w/v)]
-Sodium deoxycholate [Sigma-Aldrich, Cat.#30970, final 15% (w/v)]
-Urea [Sigma-Aldrich, Cat.#U5378, final 25% (w/v)]

Hydrogel solution (according to (Gordillo et al. 2020)):
-200 µl of 30% acrylamide–bisacrylamide (29:1) (Bio-Rad, Cat.#1610158)
-2 µl of 10% Ammonium Persulfate (APS, Sigma, A-6761)
-197 µl of H_2O
-1 µl of TEMED (Thermo Scientific, Cat.#17919). Note the addition of TEMED should only be done when the sample is ready to be embedded.

Hybridization buffer:
-100 mg/ml dextran sulphate(Sigma, Cat.#D8906-10G)-10% formamide (Milipore, Cat.#S4117)
-2x SSC (Invitrogen, Cat.#AM9763)

SCRI Renaissance 2200 staining solution
0.1% (v/v) SR2200 (stock solution of the supplier is considered as 100%)
1% (v/v) DMSO (Carl-Roth, Cat.#7029.2)
0.05% (w/v) Triton-X100
5% (w/v) glycerol (for example: SIGMA-ALDRICH, Cat.#G5516)
4% (w/v) paraformaldehyde
Note: SR2200 can be ordered from: Renaissance Chemicals Ltd, Unit 1 Blackwood Hall Business Park, North Duffield, Selby, UK. Contact: Howard Weaver, enquiries@renchem.co.uk.

Stellaris Wash Buffer A
10% (vol./vol.) formamide in 1X Stellaris RNA FISH, 5X Wash Buffer A (Biosearch Technologies Cat# SMF-WA1-60)

Wash buffer B (homemade)**:**
-10% formamide
-2x SSC

TableS1. smFISH probe sequences used in this study and the corresponding dyes (Quasar570 or Qusar670). Probe Sequences (5’-3’).

Probe nr	*PP2A* (Quasar570)	*Venus* (Quasar570)	*AtGAPDH* (Quasar670)	*HvGAPDH* (Quasar570)
1	ccgagcgatctatcaatcag	cggtgaacagctcctcgc	tgcacgcaagactgcaacg	ggttttataaagccaggagc
2	gacatcctcaccaaaactca	cagctcgaccaggatggg	actctaatcctatgggctg	agaggtggggagggtttaag
3	tcgggtataaaggctcatca	ttgtggccgtttacgtcg	gcgcaggctgatggaatat	agtgtctggaagcggatatg
4	tagctcgtcgataagcacag	ctcgccggacacgctgaa	ggggaaactaatggcgctt	cgaaggagtggaactgacgc
5	ccaagagcacgagcaatgat	ttgccgtaggtggcatcg	tcgtttatgggtaggatta	cgatcttaatcttgcccatg
6	atcaactcttttcttgtcct	gtggtgcagatcagcttc	ttatttaccatttttaccc	atccttccgaaaccgttgat
7	catcgtcattgttctcacta	agggtggtcacgagggtg	ttcttgggaaaaaggctaa	tgatgaaggggtcgttgacg
8	atagccaaaagcacctcatc	aagcactgcaggccgtag	gatcgagtggctgagattt	catgtacgtcatgtactcgg
9	atacagaataaaaccccca	catgtggtcggggtagcg	taaaagagaagaagaacgt	cgtgaactgtgtcgtacttg
10	caagtttcctcaacagtgga	tgaagaagtcgtgctgct	ttggtgaggatttagggtt	atgtcactgtgcttccaatg
11	tcatctgagcaccaattcta	acgtagccttcgggcatg	tagtgagtgagggtttggg	agcgtcttgtcgtctttgag
12	tagccagaggagtgaaatgc	agaagatggtgcgctcct	tcgagagagaagagaatgt	cctgacgccaaagacagtaa
13	cattcaccagctgaaagtcg	tcttgtagttgccgtcgt	gccattttgcgaaattgag	gtggactccacaacgtaatc
14	ggaaaatcccacatgctgat	tcgaacttcacctcggcg	ccgattctgatcttcttgt	ttgtccttgtcagtgaagac
15	atattgatcttagctccgtc	ctcgatgcggttcaccag	attcttccgaaaccgttga	cagagatgaccaccttcttg
16	attggcatgtcatcttgaca	tgaagtcgatgcccttca	ctctagcaaccaaacgacc	aaacataggggcatctttgc
17	aaattagttgctgcagctct	caggatgttgccgtcctc	ctcaacatcatccctctga	ttgtcctcattgacaccaac
18	gctgattcaattgtagcagc	ttgtactccagcttgtgc	ggatcgttaacagcgacga	atgttaacatccgaggtgta
19	ccgaatcttgatcatcttgc	agacgttgtggctgttgt	tcatgtactcggtggtgat	gtgcagctagcatttgagac
20	caaccctcaacagccaataa	tgcttgtcggcggtgata	ctgaccgtgaacactgtca	taatgaccttagctaggga
21	ctccaacaatttcccaagag	gaagttggccttgatgcc	ttaagctcatggtgcttcc	tcagaccctcaataatacca
22	caaccatataacgcacacgc	cgatgttgtggcggatct	gagttttgtcatccttcac	tgatggcatgaacagtggtc
23	agtagacgagcatatgcagg	gtagtggtcggcgagctg	tggcttctcaccgaagaga	catcaacggtcttctgtgtg
24	gaacttctgcctcattatca	cgatgggggtgttctgct	cctgatgccgaaaacagtg	gctggggatgatgttaaagc
25	cacagggaagaatgtgctgg	ttgtcgggcagcagcacg	catgggatgtcctcagggt	cattcagctcaggaagaacc
26	tgacgtgctgagaagagtct	ggactggtagctcaggta	caacaaagtcagctccagc	ccggaaagacataccggtaa
27	cccattataactgatgccaa	ttggggtctttgctcagg	tgtcagtgaagacaccagt	aactgacacatccacagtgg
28	tggttcacttggtcaagttt	catgtgatcgcgcttctc	caagtgagcagcagccttg	cggttctaacagtgagatca
29	tctacaatggctggcagtaa	ggtcacgaactccagcag	caaccttttagcaccacc	catcatatgatgcagccttc
30	cgattatagccagacgtact	tccatgccgagagtgatc	tttgcttggggcagagatg	ccttgatagccttcttgatg
31	gactggccaacaagggaata		acaacgaacatgggcgcat	catgatacccttgagctttc
32	catcaaagaagcctacacct		tactcgtgctcgttgacac	accaaatcttcctcaacgta
33	ttgcatgcaaagagcaccaa		acaatgtcaaggtcagact	tgtcaccaacgaagtcggtg
34	acggattgagtgaaccttgt		ttagtggtgcaactagcgt	agccttagcatcaaagatgc
35	cttcagattgtttgcagcag		ccttggcaagaggagcaag	cgaaatggtcgttcagagca
36	ggaccaaactcttcagcaag		gccaaacctgtcattaata	tgtcataccacgagacaagc
37	ggaactatatgctgcattgc		gtcatgagtccctcaacaa	cgacaacacggttgctgtaa
38	gtgggttgttaatcatctct		gcagtgatagagtggacag	ggaaagcagaacgctctact
39	tgcacgaagaatcgtcatcc		accatcaactgtcttctga	catagacaaaggggcacctc
40	ttactggagcgagaagcgat		ctccagtccttcattgatg	gacaccatccacatttattc
41	gaacatgtgatctcggatcc		tgaaggaagcagctcttcc	aacatgaaccaggcgtctag
42	ctctgtctttagatgcagtt			cagatggtaactcatgtcca
43	catcattttggccacgttaa			acttgactagcaactcggtg
44	cgtatcatgttctccacaac			cacttactcaggcaaacaga
45	atcaacatctgggtcttcac			caaacacttactcaggctgt
46	ttggagagcttgatttgcga			ggatcgcagcatcaaacatc
47	acacaattcgttgctgtctt			gacgggagtagtactcaaca
48	cgcccaacgaacaaatcaca			cgctccgtcctataatataa

Table S2. ClearSee incubation time for different *A.thaliana* tissues.

Tissues	Incubation time
Root	≥24hr
Young leaf	≥5days
Shoot meristem	≥5days
Inflorescence meristem	≥7days
Embryo (different stage seeds)	≥7days
Ovule (isolated)	≥4days
Young siliques	≥7days
Young buds	≥5days

Table S3. WM-smFISH strengths and limitations.

**Strengths**	• Detection and visualization of single RNA molecules allow quantification of gene expression per cell.<br>• Subcellular localization of mRNAs.<br>• Can be combined with fluorescent reporters and immunofluorescence for analysis of RNA and protein expression and subcellular colocalization in single cells.<br>• Can be readily applied in any genetic background to analyse endogenous RNA.<br>• Can be applied to non-model species.<br>• Easy to implement and does not require any advanced equipment (works well on conventional laser scanning confocal microscopes).
**Limitations**	• Probes are 18-22 mers DNA oligonucleotides and a minimum of 25-30 probes per target are required. Thereby, the target mRNA should be at least 600 bp long.<br>• smFISH signals can be harder to detect than regular immunostaining signals due to the intrinsically low signal from each transcript and its near-diffraction limit size. Therefore, WM-smFISH on tissues with high autofluorescence levels (such as green tissues) can still be challenging and requires long clearing treatments (see Table S2). Additionally, signal-to-noise can be improved by selecting fluorophores emitting at wavelengths where the tissue autofluorescence is lower.<br>• In the case of genes with extremely low expression levels, (i.e., just one or two mRNAs in a few cells only) the detection of false-positives could become significant. RNAse controls should then be considered.<br>• Due to fluorophore limitations (i.e., only a small number of colors can be used for microscopy). While we have not tried in plants smFISH has been used to study simultaneously up to four genes (Orjalo and Johansson 2016)<br>• Samples are fixed. Therefore, it cannot be used for temporal analysis of gene expression in the same cell (i.e., time-lapse or live imaging).

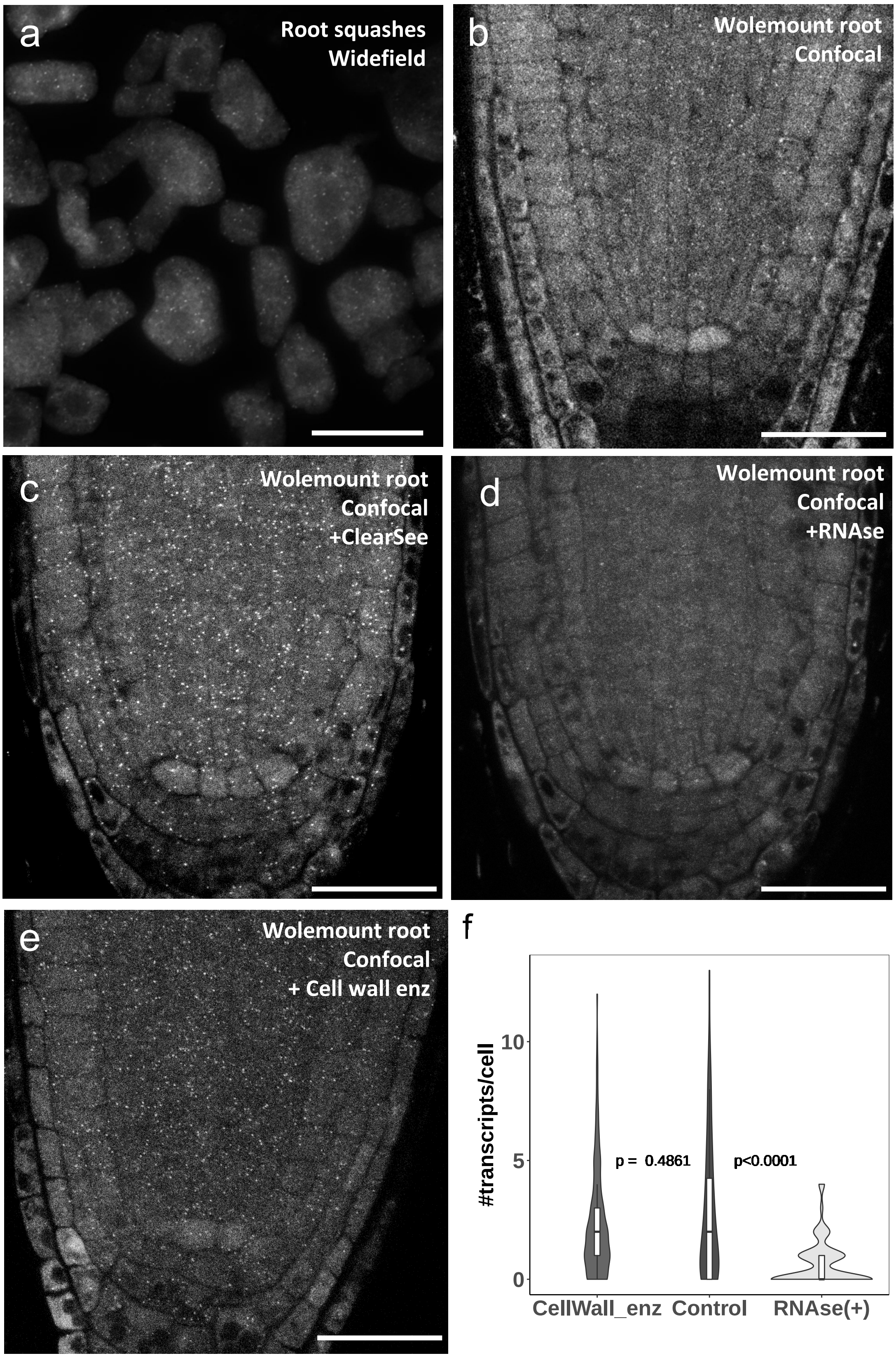

Figure S1. Optimization of tissue clearing step for whole-mount smFISH. (**a-e**) smFISH using probes against *PP2A* mRNA (grey spots). (**a**) Representative image of smFISH in root meristem squashes of 7-days-old Arabidopsis seedlings. Imaging was performed using a widefield microscope. Scale bar, 30µm. (**b-e**) Representative images of optical confocal slices of whole-mount smFISH in the root meristem of 7-days-old Arabidopsis seedling. Imaging performed using a confocal microscope. (**b**) Whole-mount smFISH in the root meristem without ClearSee treatment. (**c**) Whole-mount smFISH in the root meristem after ClearSee treatment. (**d**) Whole-mount smFISH of the root depicted in c after 15 minutes of RNase treatment. Scale bars, 30µm. (**e**) Whole-mount smFISH in the root meristem after ClearSee treatment and cell wall enzyme digestion. (**f**) Violin plot comparing the number of transcripts detected before [Control, panel C], and after treatments with RNAse [RNAse(+), panel d] cell wall enzyme digestion [CellWall_enz, panel e]. Boxes inside show the interquartile range (IQR 25-75%), indicating the median values as a horizontal line. Whiskers show the ±1.58xIQR value. A two-sided t-test was performed to compare each treatment with the control, the p-values are indicated on the graph: Control *vs* cell wall enzyme digestion (p=0.4861), Control *vs* RNAse treatment (p<0.0001). n = 130 cells Control, 130 cells RNAse(+), 166 cells CellWall_enz. Experiments were repeated independently 2 times.

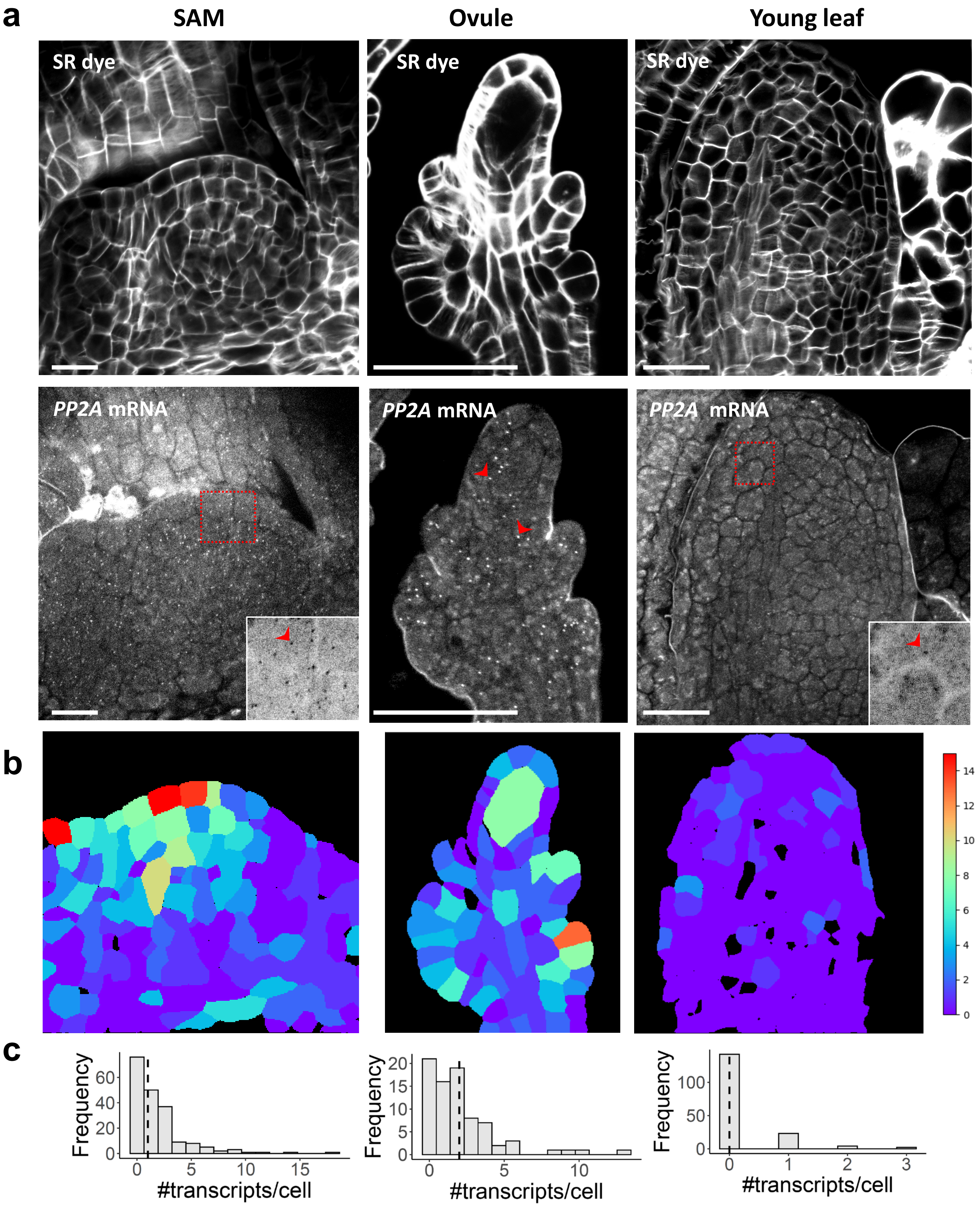

Figure S2. WM-smFISH in other Arabidopsis tissues using *PP2A* probes. (**a**) Representative images of whole-mount smFISH in Arabidopsis shoot apical meristem (SAM, left images); ovule (middle images); young leaf (right images). Top row images: cell wall stained with Renaissance 2200 (SR dye); middle row images: *PP2A* smFISH channel, insets show inverted zoomed-in image. Red arrows point to mRNA dots. Scale bars, 10μm. (**b**) Heatmaps representing the number of transcripts per cell detected for the SAM (left), ovule (middle), young leaf (right). (**c**) Histograms showing the distribution of the number of transcripts per cell for the SAM (left), ovule (middle), young leaf (right). The median value is indicated with a dashed line. Experiments were repeated independently 4 times.

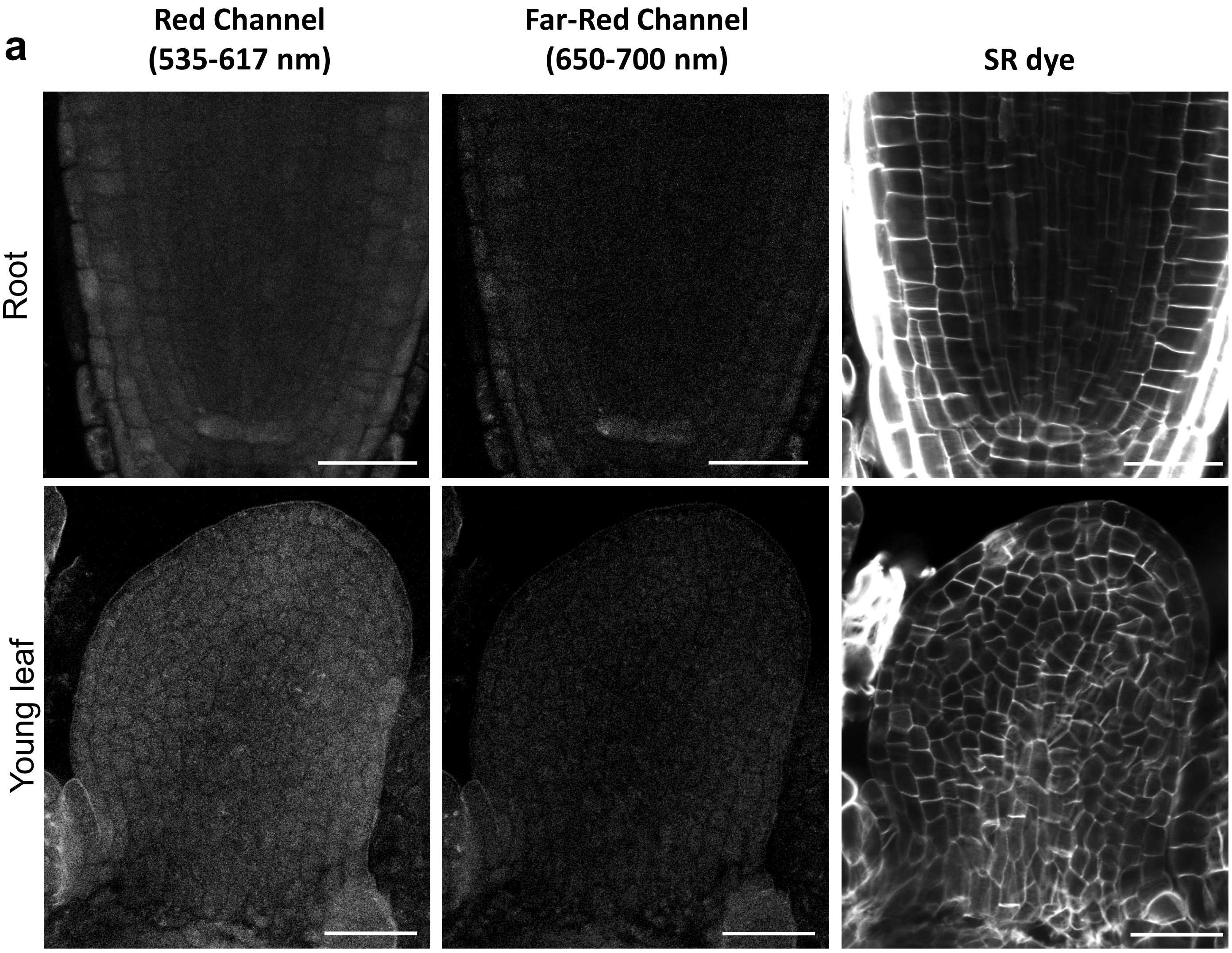

Figure S3. Controls for background signal in root and young leaves. (a) Representative confocal images of no-probe smFISH controls for root and young leaf showing background signal at red (535-617 nm) and far-red (650-700 nm) channels. Scale bars, 30µm. Experiments were repeated independently 2 times.

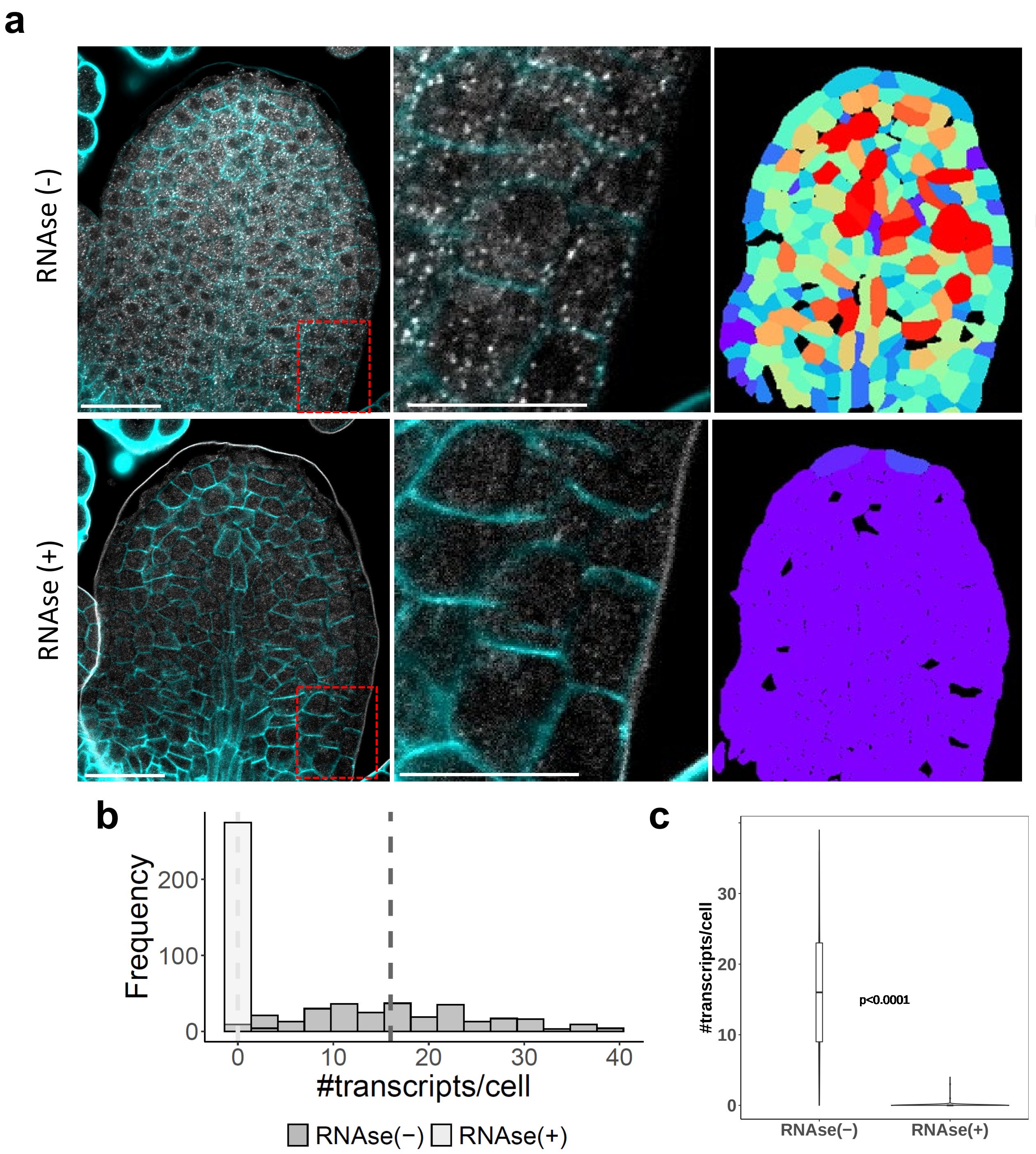

Figure S4. WM-smFISH in Arabidopsis young leaves using *GAPDH* probes. (**a**) Representative images of whole-mount smFISH using probes targeting *GAPDH* mRNA in young leaf before (top panels) and after RNAse treatment (bottom panels). Left panels: merged images with *GAPDH* smFISH channel (grey) and cell wall dye (cyan); Scale bars, 30µm. Middle panels: zoomed-in images from the regions highlighted in red on the left panel; Scale bars, 10µm. Right panel: Heatmaps representing the number of transcripts per cell. (**b**) Histogram showing the distribution of the number of transcripts per cell before and after RNAse treatment. The median value is indicated with a dashed line. (**c**) Violin plot comparing the number of transcripts detected before RNAse (-) and after RNAse (+) treatment (n = 292 cells). Boxes inside show the interquartile range (IQR 25-75%), indicating the median values as a horizontal line. Whiskers show the ±1.58xIQR value. A two-sided t-test was performed to compare both conditions, the p-value is indicated on the graph ($p<0.0001$). Experiments were repeated independently 2 times.

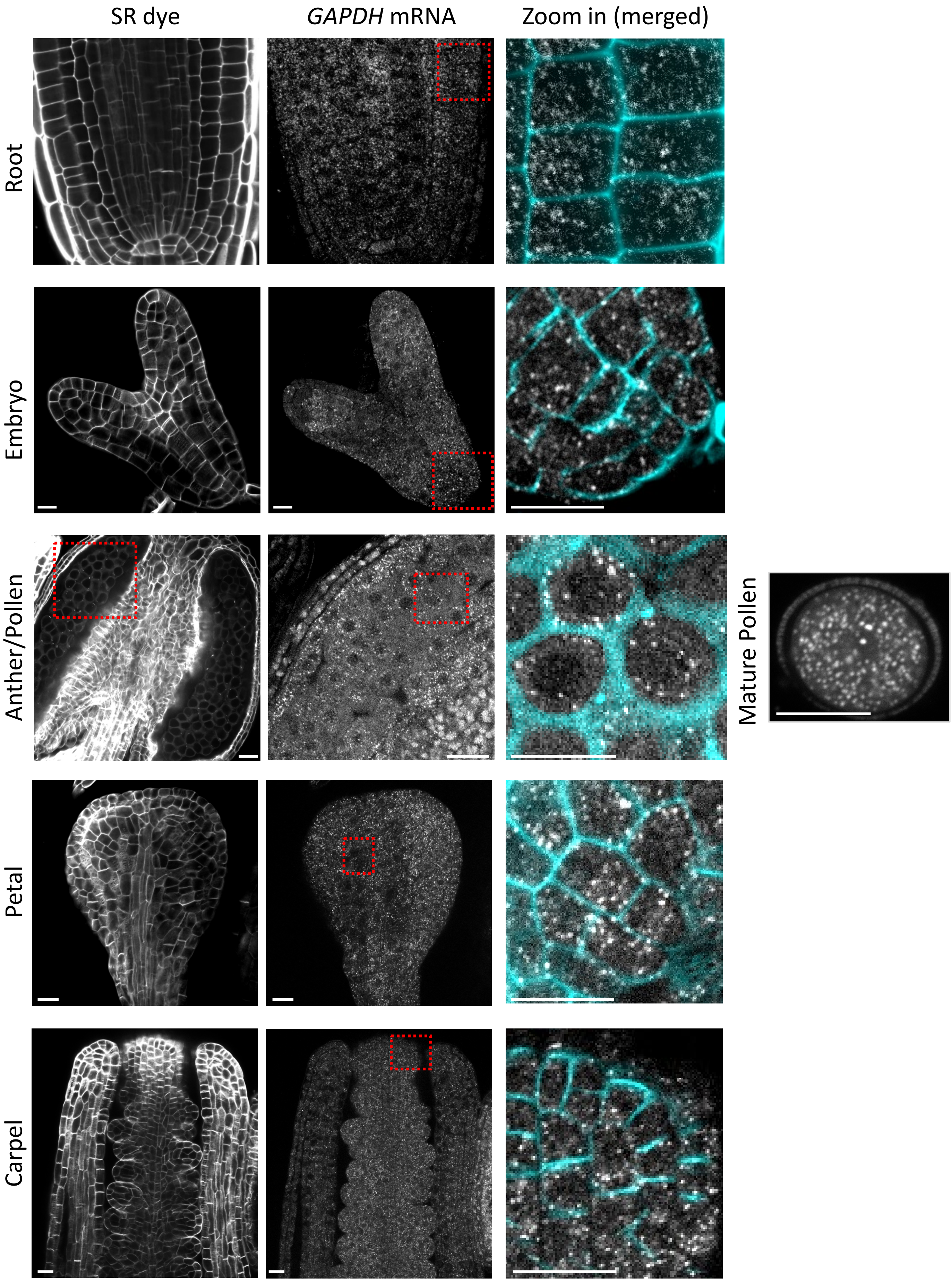

Figure S5. WM-smFISH in other Arabidopsis tissues using *GAPDH* probes. Representative images of whole-mount smFISH in Arabidopsis root, embryo, anther/pollen, petal and carpel. Left panels: cell wall stained with Renaissance 2200 (SR dye). Middle panels: *GAPDH* smFISH channel. Right panels: merged zoomed-in images from the regions highlighted in red on the middle panel; smFISH GAPDH signal (grey) and cell wall dye (cyan). An image of a mature pollen is also shown (*GAPDH* smFISH channel only). Scale bars, 10µm. Experiments were repeated independently 2 times.

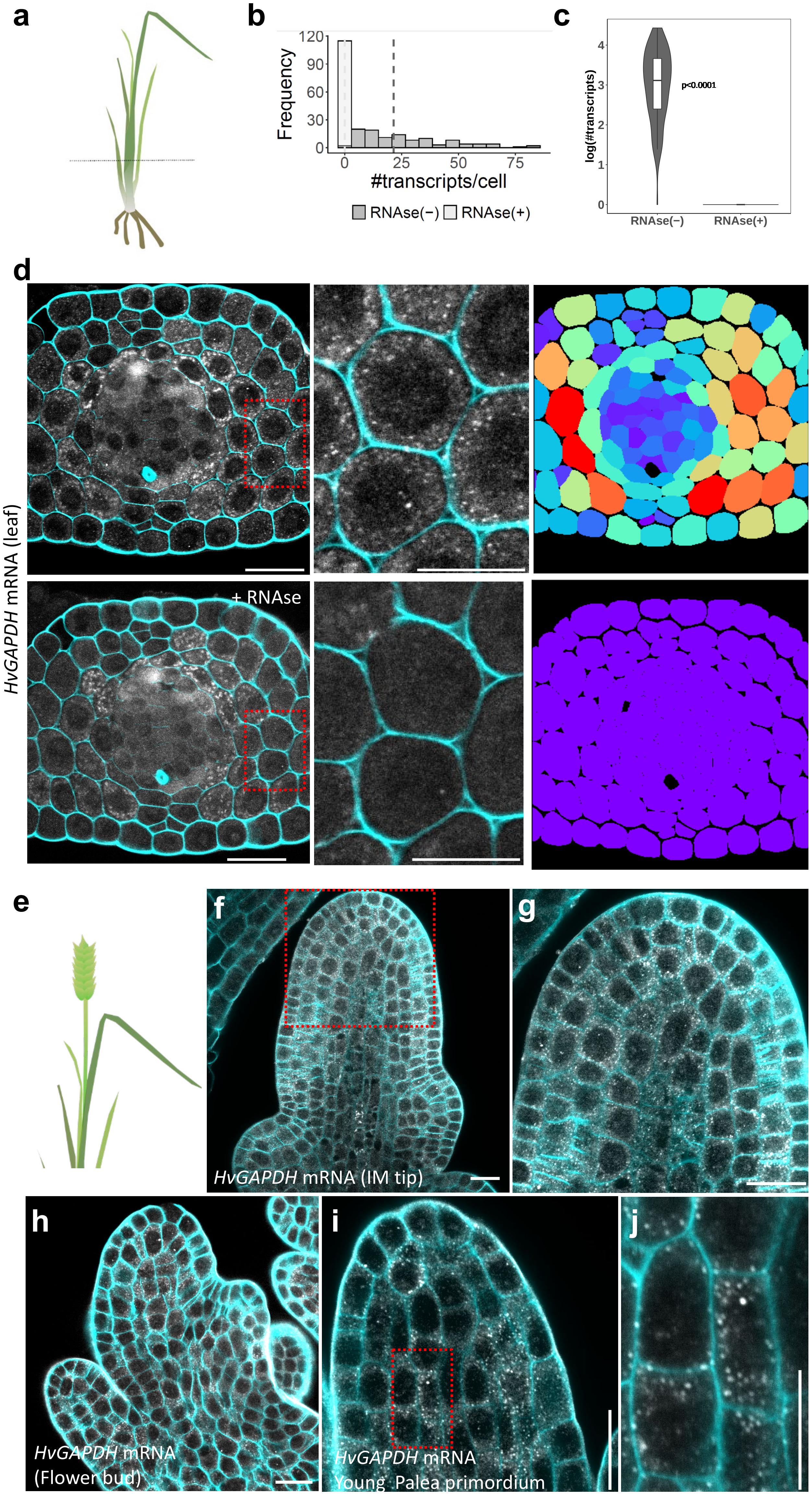

a
b
Frequency
120
90
60
30
0
0
25
50
75
#transcripts/cell
RNAse(−)
RNAse(+)
c
log(#transcripts)
4
3
2
1
0
p<0.0001
RNAse(−)
RNAse(+)
d
HvGAPDH mRNA (leaf)
+ RNAse
RNA/
cell
70
60
50
40
30
20
10
0
e
f
g
HvGAPDH mRNA (IM tip)
h
i
j
HvGAPDH mRNA
(Flower bud)
HvGAPDH mRNA
Young Palea primordium

Figure S6. WM-smFISH in barley (*Hordeum vulgare*) leaf using *GAPDH* probes. (**a**) Schematic representation of barley seedling showing the plane of dissection. (**b**) Histogram showing the distribution of the number of transcripts per cell with (+) and without (-) RNAse treatment. The median value is indicated with a dashed line. (**c**) Violin plot comparing the number of transcripts detected before RNAse (-) and after RNAse (+) treatment Boxes inside show the interquartile range (IQR 25-75%), indicating the median values as a horizonal line. Whiskers show the $\pm$1.58xIQR value. A two-sided t-test was performed to compare both conditions, the p-value is indicated on the graph (n = 113 cells, p<0.0001). (**d**) Representative images of whole-mount smFISH in barley leaf without RNAse (top panels) and with RNAse (bottom panels) treatment. Left panels: merged images with *GAPDH* smFISH channel (grey) and cell wall dye (cyan); Scale bars, 20µm. Middle panels: zoomed-in images from the regions highlighted in red on the left panel; Scale bars, 10µm. Right panel: Heatmaps representing the number of transcripts per cell. (**e**) Schematic representation of mature barley inflorescence. (**f-j**) Representative merged images of whole-mount smFISH in barley inflorescences with *GAPDH* smFISH channel (grey) and cell wall dye (cyan). Scale bars, 20µm; except for **j** in which scale bar = 10µm. The apex of a young inflorescence (**f**), a young flower primordia (**h**) and a developing palea (**i**) are shown. Magnified images of the regions highlighted in red in **f** and **h** are shown in **g** and **j** respectively. Experiments were repeated independently 2 times.

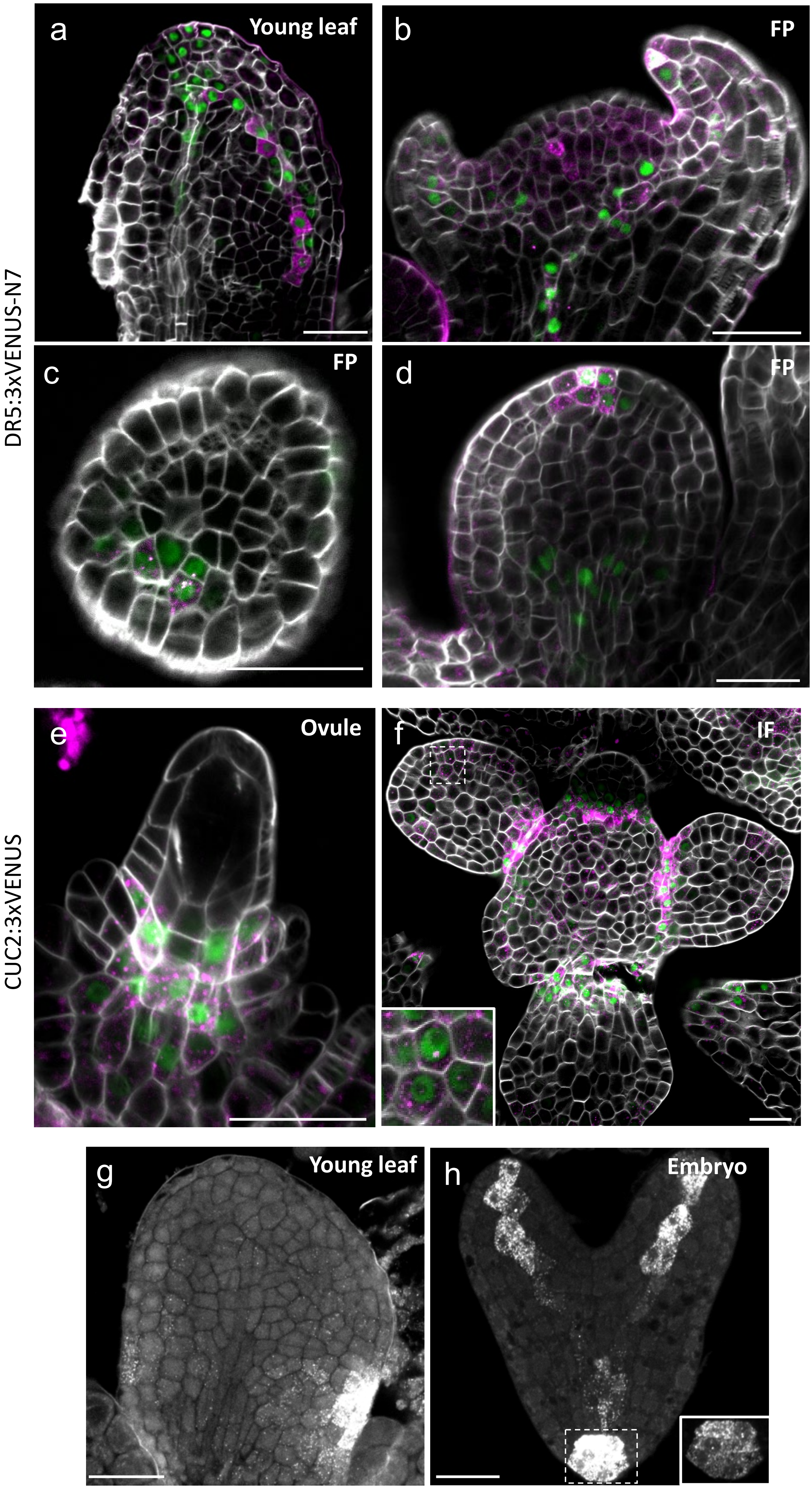

Figure S7. WM-smFISH for simultaneous detection of mRNA and protein in different tissues. (**a-f**) Representative images for WM-smFISH for the detection of VENUS mRNA (magenta) and protein (green) in *pDR5rev::3xVENUS-N7* and *pCUC2::3xVENUS-N7* reporter lines in: young leaf (**a**); floral primordia (**d-d**); ovule (**e**); inflorescence meristem (**f**). (**g-h**) WM-smFISH images depicted in Figure 2e (embryo) and 2g (young leaf) showing only the smFISH probe channel in grey. Inset (**h**): showing the same region with lower brightness and contrast levels, allowing to observe single dotted signals corresponding to mRNAs. Scale bars, 20µm. Experiments were repeated independently at least 3 times.

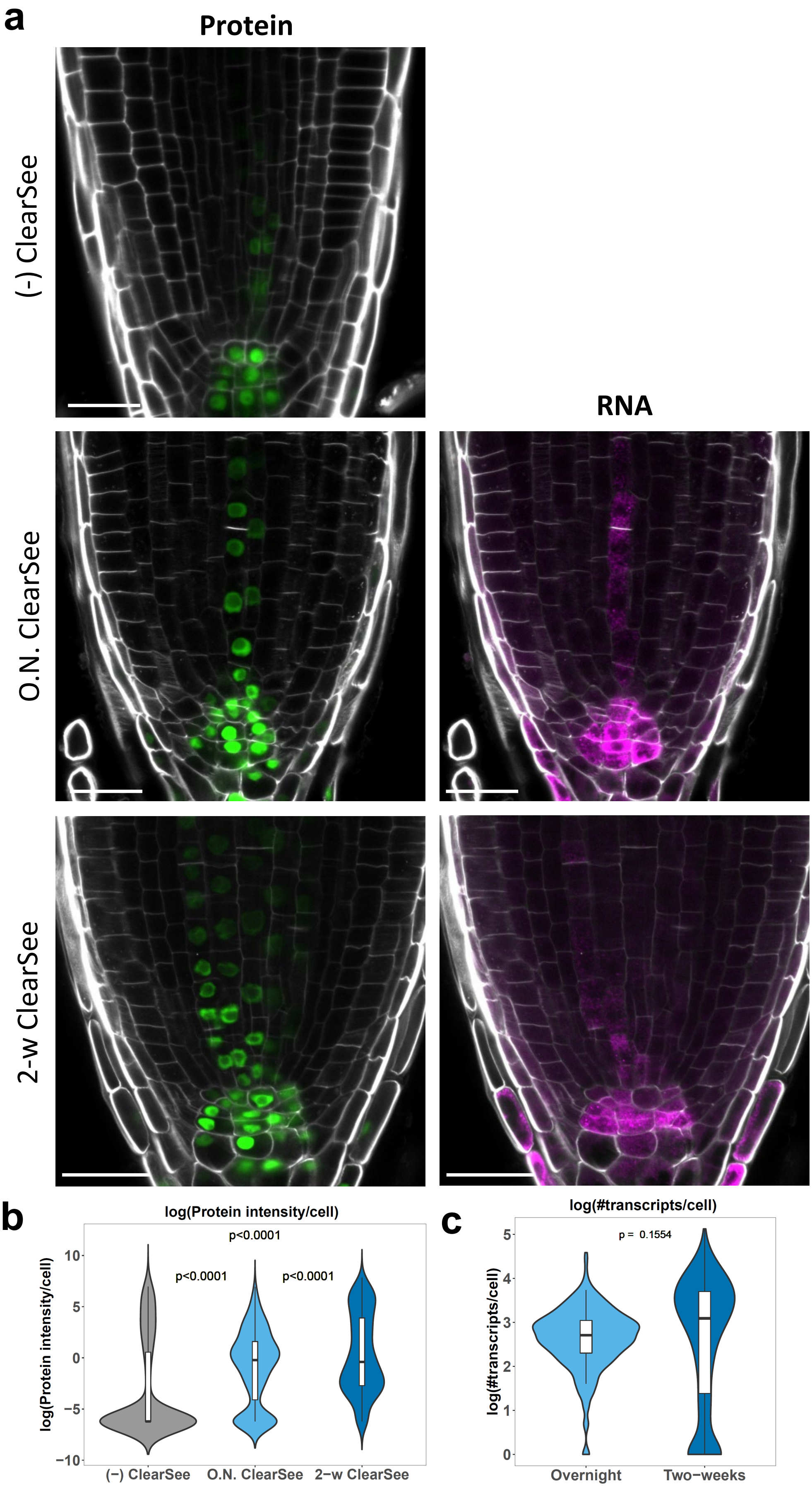

Figure S8. Quantification of fluorescent protein signal and mRNA smFISH counts after ClearSee treatment. (**a**) Representative confocal images of 7-day-old Arabidopsis roots expressing *pDR5rev::3xVENUS-N7* reporter lines showing VENUS fluorescent protein detection (green) and WM-SmFISH mRNA signals (magenta) for roots without ClearSee treatment (-) and roots treated overnight (O.N.) and two weeks (2-w) with ClearSee. The contours of cells were visualized through cell wall staining with Renaissance 2200 (white). Scale bars, 20µm. (**b-c**) Violin plots showing the distributions for (**b**) total protein intensity per cell and (**c**) the number of transcripts detected in all the treated roots [(-)ClearSee: 819 cells, Overnight/O.N Clearsee: 788 cells, Two-weeks/2-w Clearsee: 802 cell; from 5 roots per condition]. Values are on a natural logarithmic scale. Boxes inside show the interquartile range (IQR 25-75%), indicating the median values as a horizontal line. Whiskers show the ±1.58xIQR value. The p-values for ANOVA followed by a one-sided TukeyHSD test are shown for all the pair comparisons in **(b)**: (-)ClearSee *vs* O.N Clearsee (p<0.0001), (-)ClearSee *vs* 2-w Clearsee (p<0.0001), O.N ClearSee *vs* 2-w Clearsee (p<0.0001). The p-value for a two-sided t-test is shown in **(c)** (p=0.1554). Experiments were repeated independently 2 times.

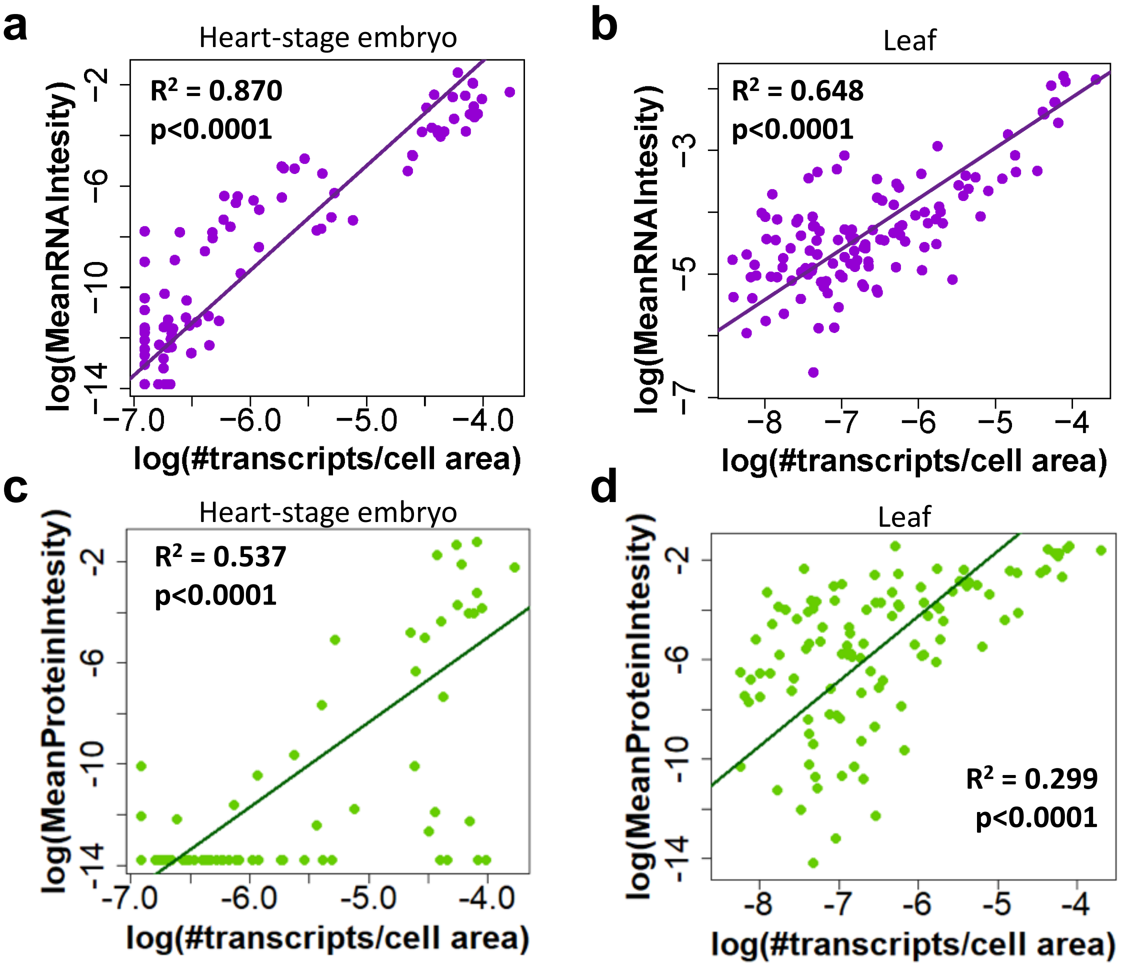

Figure S9. Correlation between the number of transcripts per cell and the total fluorescence intensity of RNA or proteins at the cellular level. Scatter plots showing the correlation between the number of transcripts per cell area and the mean RNA intensity (by smFISH) (**a**, **b**), or protein intensity (**c**, **d**) per cell (in logarithmic scales) detected for the representative images shown in Figure 2. A linear regression was calculated between the two variables for each plot. The determinant coefficient (R^2) and adjusted p-value of each regression are indicated **(a)** R^2 = 0.870, p<0.0001, b**)** R^2 = 0.648, p<0.0001, **c)** R^2 = 0.537, p<0.0001, **a)** R^2 = 0.299, p<0.0001. (**a**, **c**) Heart-stage embryo. (**b**, **d**) Leaf. Experiments were repeated independently at least 3 times.

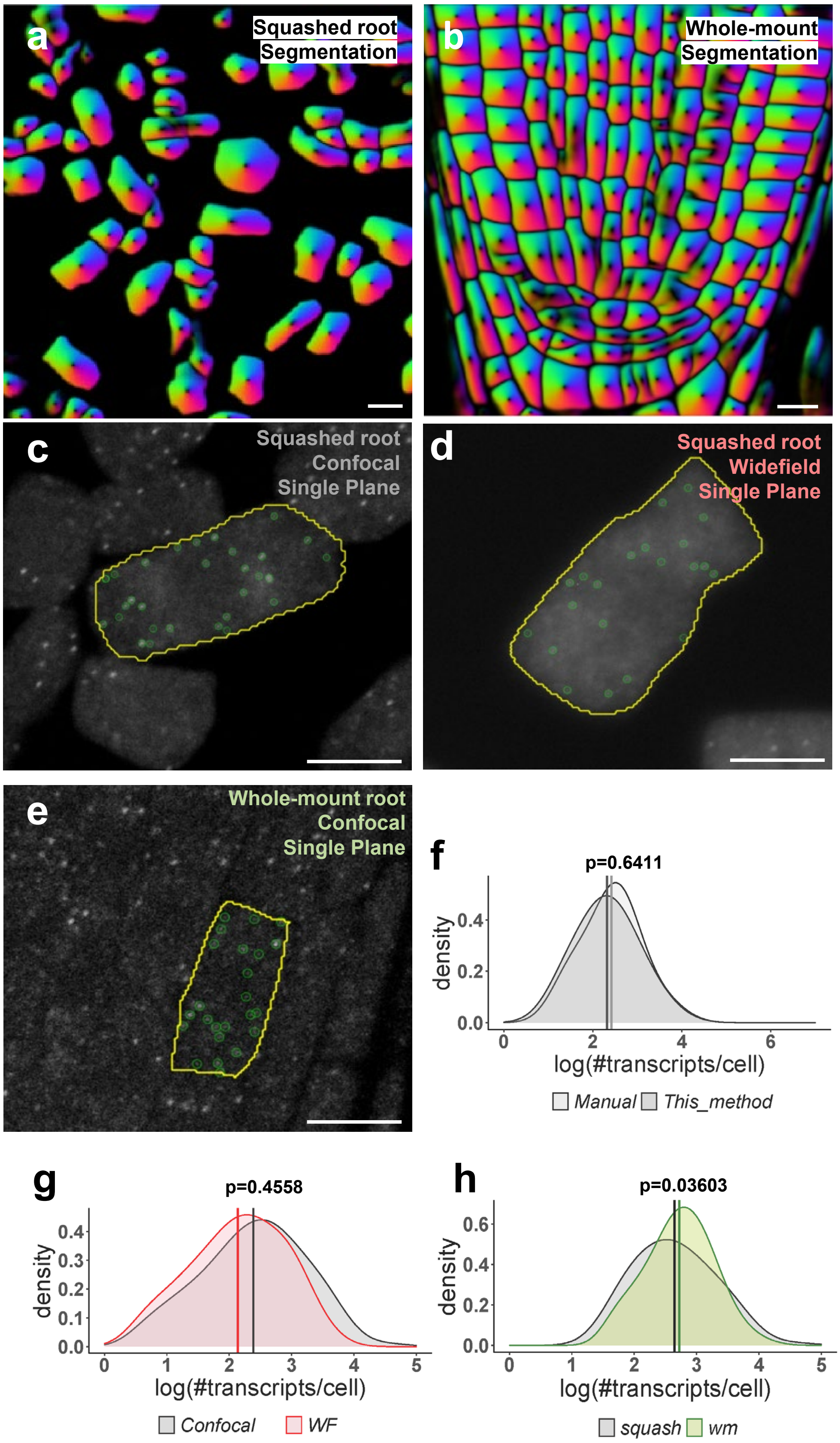

Figure S10. Evaluation of the RNA-single molecule quantification workflow in images acquired by different methods. (**a-b**) Representative images showing the cell segmentation results obtained for squashed-root tip cells (**a**) and from a whole-mount root (**b**) using Cellpose. (**c-e**) Representative images showing the detected RNA molecules by FISHquant using different acquisition methods: (**c**) squashed root, confocal microscopy, single plane; (**d**) squashed root, widefield microscopy, single plane; (**e**) whole-mount root, confocal microscopy, single plane. (**f-h**) Density plots comparing the distributions for the number of RNAs per cell (in natural logarithmic scale) using different methods. Distributions were statistically compared using a two-sample centered Kolmogorov-Smirnov test, p-values are indicated on the graph. (**f**) Comparison between detection by eye (manual) and the workflow from this paper (This_method) in images from a squashed root, obtained with widefield microscopy in Z-planes (N = 61 cells), p=0.6411. (**g**) Comparison between images from squashed roots using confocal or widefield (WF) microscopy (Confocal: 520 cells – 8 roots, WF: 255 cells – 6 roots), p=0.4558. (**h**) Comparison of the distributions obtained from squashed roots (squash) or whole-mount roots (wm) analyzing one plane from confocal microscopy (squash: 520 cells, wm: 1249 cells; from 8 roots per condition), p=0.03603. Scale bars, 10µm. Experiments were repeated independently at least 3 times.

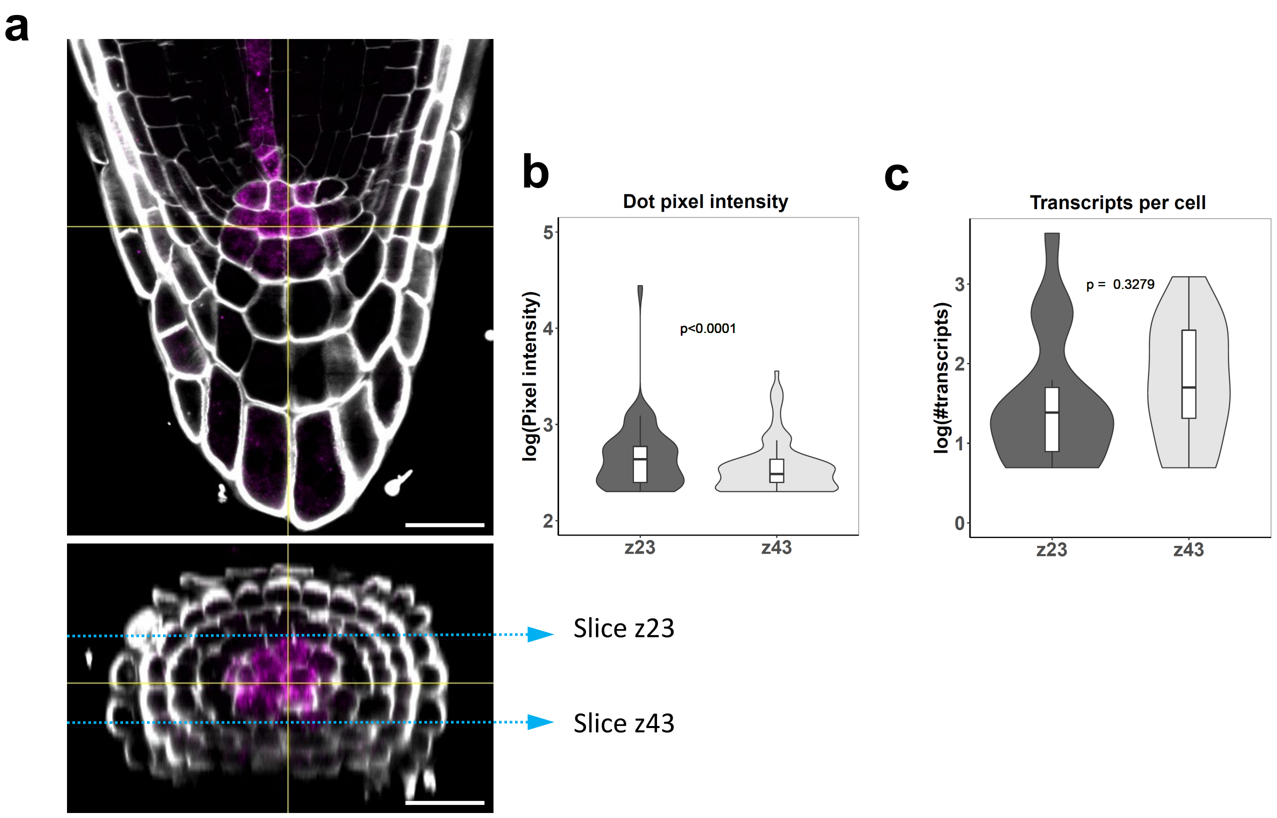

Figure S11. Quantification of *Venus* transcripts per cell in different Z-layers. (**a**) Representative image of WM-smFISH on 7-day-old Arabidopsis roots of *pDR5rev::3xVENUS-N7* line with probes against *Venus* mRNA. Top: XY view of the middle layer of the root. Bottom: XZ view of the middle layer of the root. Two Z-slices were picked for comparison: z23 and z43. (**b-c**) Violin plot comparing the transcript detection in the selected Z-layers. (**b**) Compares the pixel intensity of each detected dot (111 dots in z23, 107 dots in z43). (**c**) Compares the number of transcripts per cell detected in each slice (72 cells in z23, 68 cells in z43). Values are on a natural logarithmic scale. Boxes inside show the interquartile range (IQR 25-75%), indicating the median values as a horizontal line. Whiskers show the ±1.58xIQR value. A two-sided t-test was performed to compare both slices, the p-value is indicated on the graph: **(b)** $p<0.0001$, **(c)** $p=0.3279$. Scale bars, 20µm. Experiments were repeated independently 2 times.

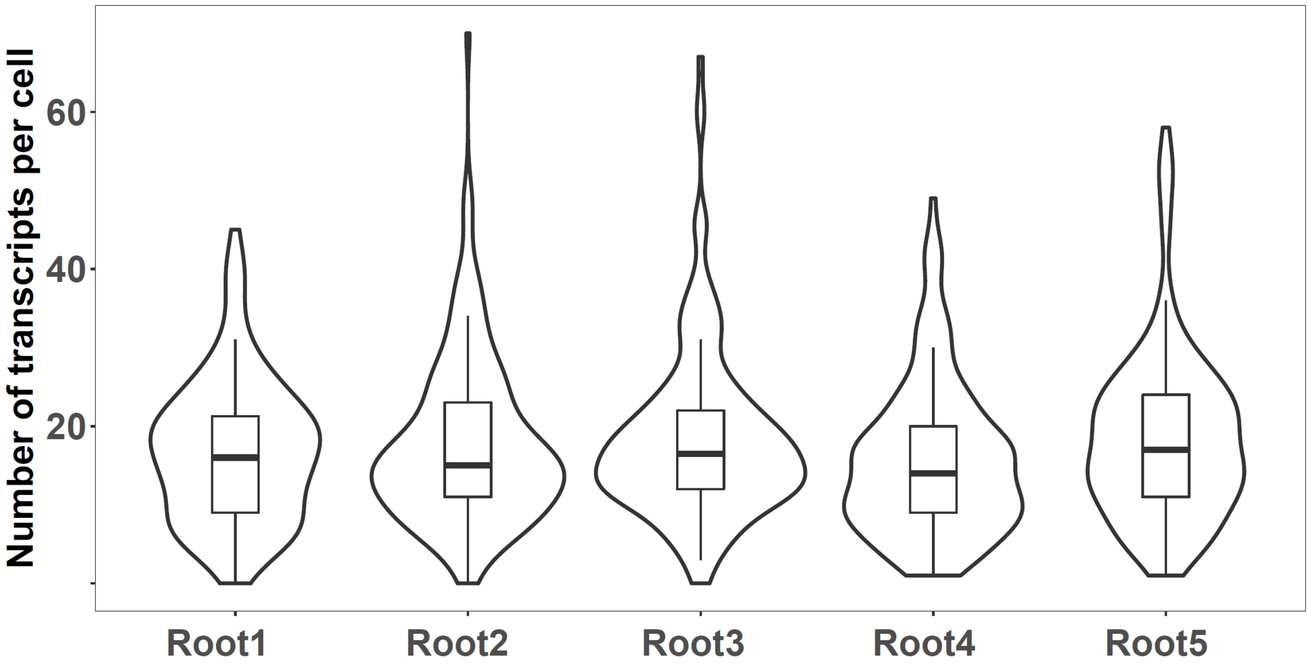

Figure S12. Quantification reproducibility for *PP2A* transcript detection. Violin plot comparing the number of *PP2A* transcripts per cell detected in the middle section of five different roots. Boxes inside show the interquartile range (IQR 25-75%), indicating the median values as a horizontal line. Whiskers show the ±1.58xIQR value. The variation associated with biological samples is relatively low as the standard deviation of the mean values per root was 1,592 transcripts/cell for a mean of 18.033 which correspond to a coefficient of variation of 0,088.

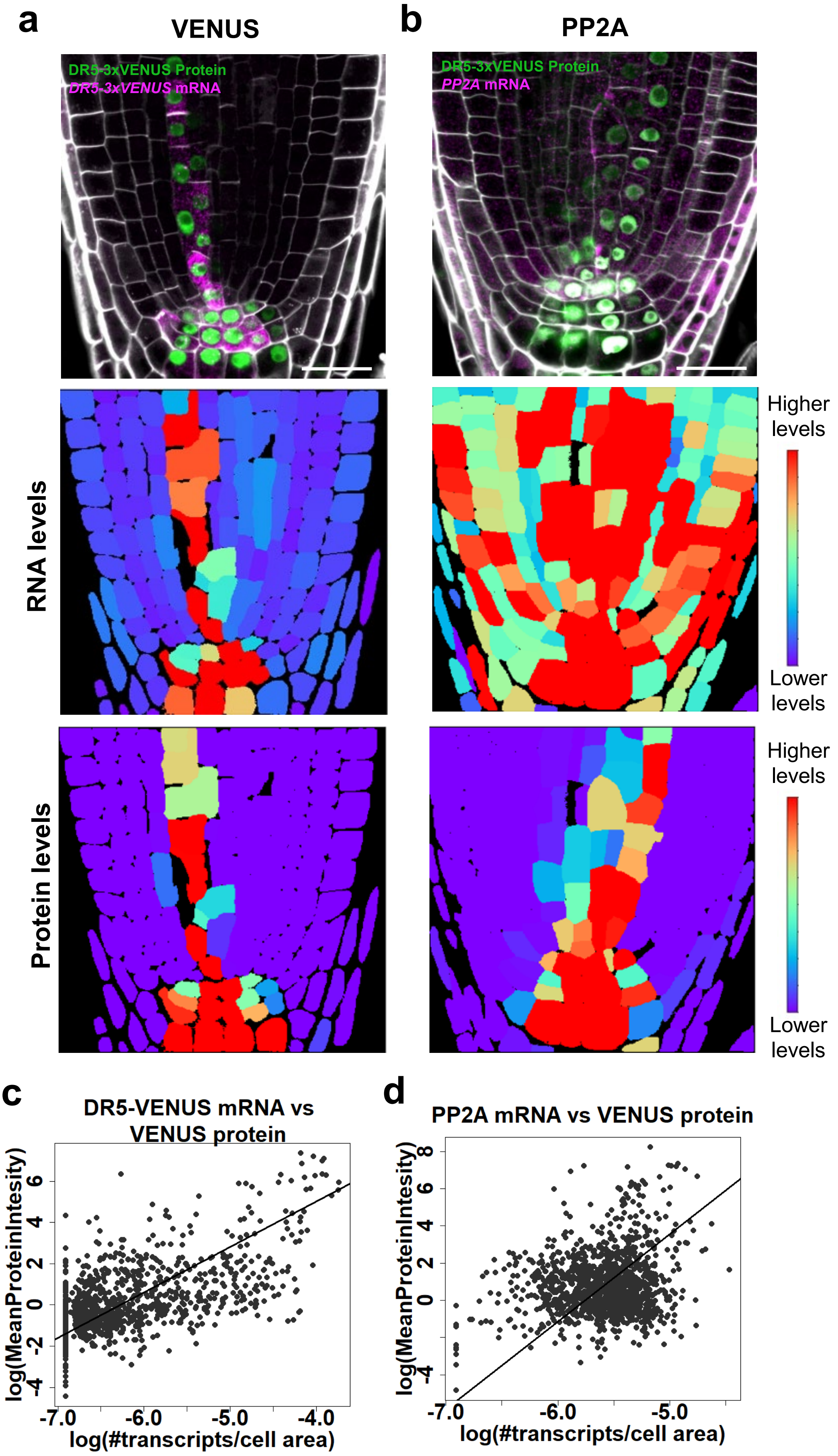

Fig.S13. Specificity detection validation for the RNA single-molecule quantification method. Representative images to quantify the *VENUS* (**a**) or *PP2A* (**b**) mRNAs in cells from the meristematic zone in roots from 7-day old *pDR5rev::3xVENUS-N7* reporter lines. Confocal images (upper panels) show the simultaneous detection of the respective mRNA (magenta), VENUS protein (green), and cell contours with Renaissance 2200 dye (white). Scale bars, 20µm. Heatmaps represent the levels of the mean signal intensity per cell detected in the channels for RNA (middle panels) or protein detection (bottom panels) in the representative images shown in the top panels. Heatmaps represent the ratio between the RNA and protein signal intensities per cell in the representative images shown in the top panels. (**c-d**) Correlation between the number of transcripts per cell area and the mean protein intensity per cell (in logarithmic scales) detected for *VENUS* or *PP2A* mRNAs. A linear regression was calculated between the two variables for each plot. The determinant coefficient (R^2) and adjusted p-value of each regression are indicated **(c)** R^2 = 0.3955, p<0.0001, **(d)** R^2 = 0.0354 (VENUS: 1136 cells, PP2A: 1299 cells ; from 9 roots per gene). Experiments were repeated independently 3 times.

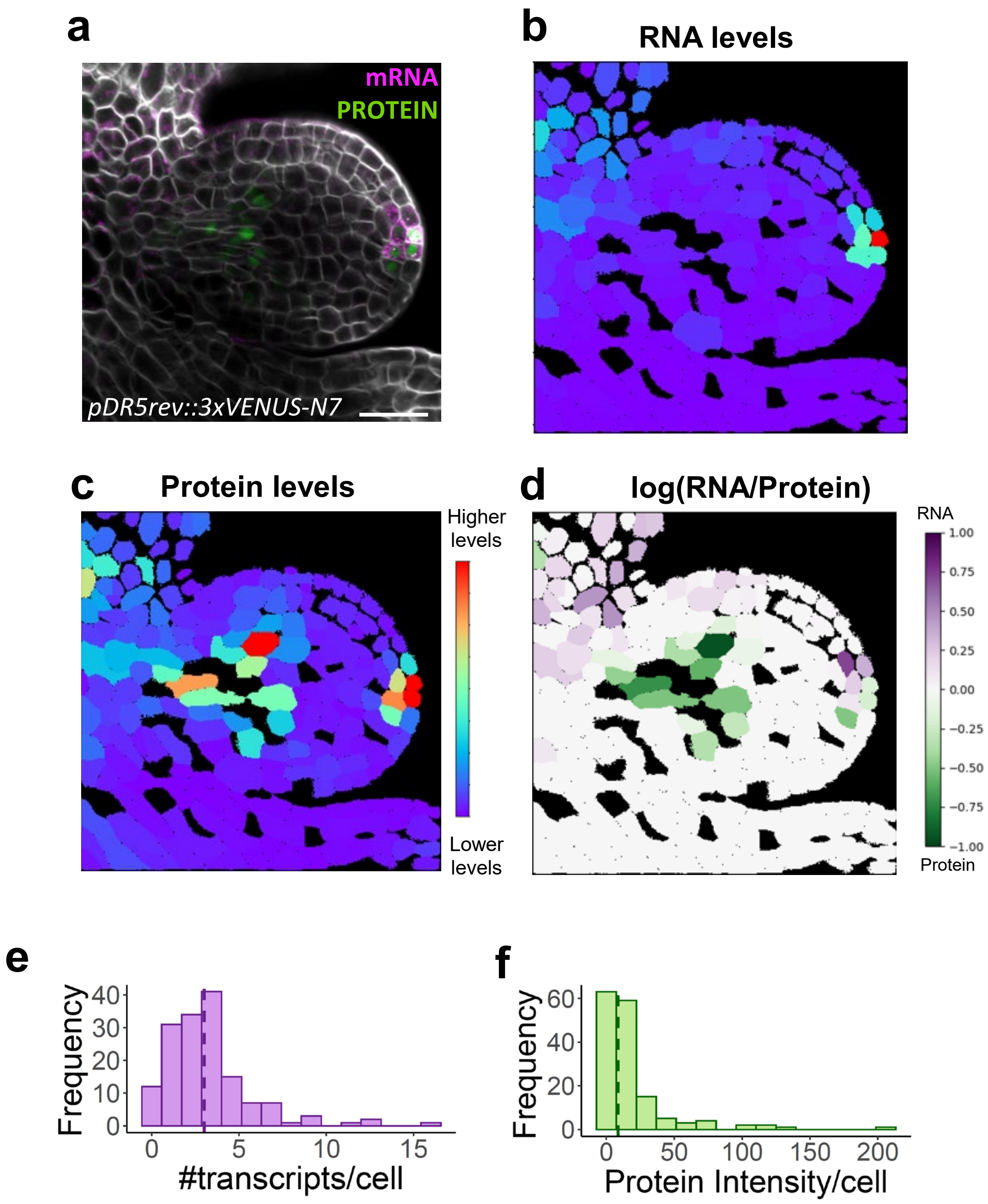

Figure S14. Simultaneous quantification for RNA and protein quantification in floral primordia. Representative image of simultaneous mRNA and protein in floral primordia using a *pDR5rev::3xVENUS-N7* reporter line. (**a**) Confocal microscopy image detecting mRNA (magenta), protein (green), and cell contours with Renaissance 2200 dye (white). Scale bars, 20µm. (**b-c**) Heatmaps represent the levels of the mean signal intensity per cell detected in each channel for RNA (**b**) or protein (**b**) detection. (**d**) Heatmap representing the ratio between the RNA and protein signal intensities per cell. (**e-f**) Histograms showing the distribution of the number of transcripts (**e**) or total protein intensity (**f**) per cell (right), the median value is indicated with a dashed line. Experiments were repeated independently 3 times.

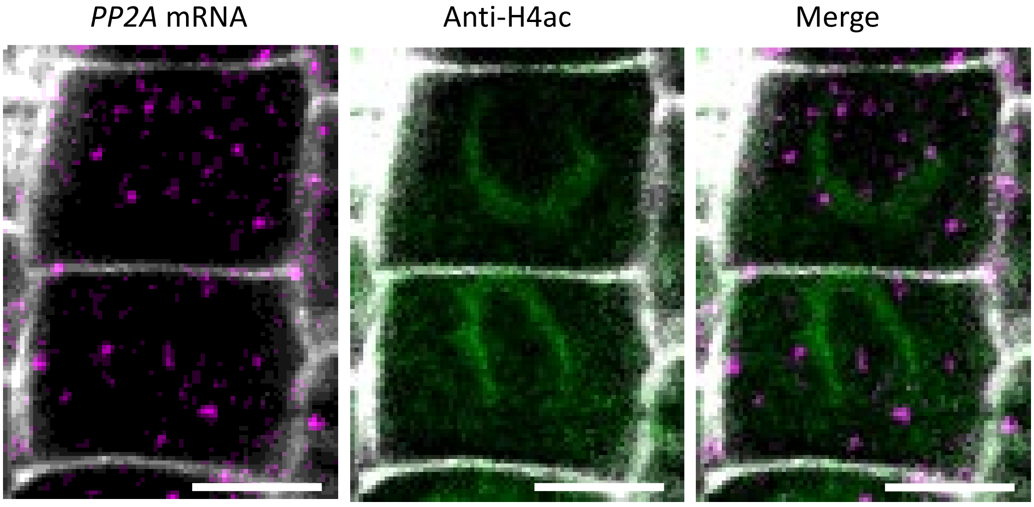

Figure S15. Sequential smFISH/Immunofluorescence. As we describe in the methods, we performed a protocol in which we combined WM-smFISH and Immunofluorescence in Arabidopsis roots, here using smFISH probes against *PP2A* mRNAs (magenta) and an antibody against acetylated histone H4. The contours of cells were visualized through cell wall staining with Renaissance 2200 (white). Scale bar, 5µm. Experiments were repeated independently 2 times.

References for supplemental material:

Duncan, S., T. S. G. Olsson, M. Hartley, C. Dean & S. Rosa (2016) A method for detecting single mRNA molecules in Arabidopsis thaliana. *Plant Methods,* 12**,** 13.

Gamborg, O. L., T. Murashige, T. A. Thorpe & I. K. Vasil (1976) Plant tissue culture media. *In Vitro,* 12**,** 473-8.

Gordillo, S. V. G., R. Escobar-Guzman, D. Rodriguez-Leal, J. P. Vielle-Calzada & A. Ronceret (2020) Whole-Mount Immunolocalization Procedure for Plant Female Meiocytes. *Methods Mol Biol,* 2061**,** 13-24.

Imbert, A., W. Ouyang, A. Safieddine, E. Coleno, C. Zimmer, E. Bertrand, T. Walter & F. Mueller (2022) FISH-quant v2: a scalable and modular tool for smFISH image analysis. *RNA,* 28**,** 786-795.

Kurihara, D., Y. Mizuta, Y. Sato & T. Higashiyama (2015) ClearSee: a rapid optical clearing reagent for whole-plant fluorescence imaging. *Development,* 142**,** 4168-79.

Mueller, F., A. Senecal, K. Tantale, H. Marie-Nelly, N. Ly, O. Collin, E. Basyuk, E. Bertrand, X. Darzacq & C. Zimmer (2013) FISH-quant: automatic counting of transcripts in 3D FISH images. *Nat Methods,* 10**,** 277-8.

Musielak, T. J., L. Schenkel, M. Kolb, A. Henschen & M. Bayer (2015) A simple and versatile cell wall staining protocol to study plant reproduction. *Plant Reprod,* 28**,** 161-9.

Orjalo, A. V., Jr. & H. E. Johansson (2016) Stellaris(R) RNA Fluorescence In Situ Hybridization for the Simultaneous Detection of Immature and Mature Long Noncoding RNAs in Adherent Cells. *Methods Mol Biol,* 1402**,** 119-134.

Ouyang, W., F. Mueller, M. Hjelmare, E. Lundberg & C. Zimmer (2019) ImJoy: an open-source computational platform for the deep learning era. *Nat Methods,* 16**,** 1199-1200.

Stirling, D. R., M. J. Swain-Bowden, A. M. Lucas, A. E. Carpenter, B. A. Cimini & A. Goodman (2021) CellProfiler 4: improvements in speed, utility and usability. *BMC Bioinformatics,* 22**,** 433.

Stringer, C., T. Wang, M. Michaelos & M. Pachitariu (2021) Cellpose: a generalist algorithm for cellular segmentation. *Nat Methods,* 18**,** 100-106.
