# Supplementary material for: Whole-mount smFISH allows combining RNA and protein quantification at cellular and subcellular resolution
Source: Nat Plants. 2023 Jun 15;9(7):1094–102. doi: 10.1038/s41477-023-01442-9 (PMC10356603; doi:10.1038/s41477-023-01442-9)
Supplement: Supplementary file 2 — Reporting Summary [file 41477_2023_1442_MOESM2_ESM.pdf]

nature portfolio

Corresponding author(s): Stefanie Rosa and Adrien Sicard

Last updated by author(s): May 2, 2023

Reporting Summary

Nature Portfolio wishes to improve the reproducibility of the work that we publish. This form provides structure for consistency and transparency in reporting. For further information on Nature Portfolio policies, see our Editorial Policies and the Editorial Policy Checklist.

Statistics

For all statistical analyses, confirm that the following items are present in the figure legend, table legend, main text, or Methods section.

n/a	Confirmed	
☐	☒	The exact sample size (*n*) for each experimental group/condition, given as a discrete number and unit of measurement
☐	☒	A statement on whether measurements were taken from distinct samples or whether the same sample was measured repeatedly
☐	☒	The statistical test(s) used AND whether they are one- or two-sided<br>*Only common tests should be described solely by name; describe more complex techniques in the Methods section.*
☐	☒	A description of all covariates tested
☒	☐	A description of any assumptions or corrections, such as tests of normality and adjustment for multiple comparisons
☐	☒	A full description of the statistical parameters including central tendency (e.g. means) or other basic estimates (e.g. regression coefficient) AND variation (e.g. standard deviation) or associated estimates of uncertainty (e.g. confidence intervals)
☐	☒	For null hypothesis testing, the test statistic (e.g. *F*, *t*, *r*) with confidence intervals, effect sizes, degrees of freedom and *P* value noted<br>*Give P values as exact values whenever suitable.*
☒	☐	For Bayesian analysis, information on the choice of priors and Markov chain Monte Carlo settings
☒	☐	For hierarchical and complex designs, identification of the appropriate level for tests and full reporting of outcomes
☐	☒	Estimates of effect sizes (e.g. Cohen's *d*, Pearson's *r*), indicating how they were calculated

Our web collection on statistics for biologists contains articles on many of the points above.

Software and code

Policy information about availability of computer code

Data collection

We used Zen Black 2.0 to record data from microscopes.

Data analysis

1) Probes directed against the genes of interest were designed using the LGC Biosearch Technologies' Stellaris® version 4.2. (https://www.biosearchtech.com/support/tools/design-software/stellaris-probe-designer).

2) The images were segmented into individual cell outlines using Cellpose 1.0 (Stringer et al., 2021). We run Cellpose with an interface using the plugin "SegmentObjects" (object type: Cells) in ImJoy v0.11.39 (Ouyang et al., 2019). This plugin and a manual for users are freely provided by Florian Mueller on Github (https://github.com/fish-quant/fq-segmentation).

3) To detect the RNA foci, we used the Matlab written software FISH-quant v3 (Mueller et al., 2013). The software and manual are provided on Bitbucket by Florian Mueller (https://bitbucket.org/muellerflorian/fish_quant/src/master/).

4) We used Cellprofiler version 4.2.1 (Stirling et al., 2021) to invert the signal of the membrane marker, quantify the protein intensity per cell, create heatmaps for cellular RNA and protein levels, and perform the colocalization analysis. All the Cellprofiler pipelines used in this work are freely available on the Cellprofiler website (https://cellprofiler.org/published-pipelines).

5) The density, violin, scatter plots, and statistical analyses were created using ggplot2 or base R-packages.

For manuscripts utilizing custom algorithms or software that are central to the research but not yet described in published literature, software must be made available to editors and reviewers. We strongly encourage code deposition in a community repository (e.g. GitHub). See the Nature Portfolio guidelines for submitting code & software for further information.

Data

Policy information about availability of data

All manuscripts must include a data availability statement. This statement should provide the following information, where applicable:

- Accession codes, unique identifiers, or web links for publicly available datasets
- A description of any restrictions on data availability
- For clinical datasets or third party data, please ensure that the statement adheres to our policy

We have made all the microscopy images available through figshare. We have added the following statement: "All the raw microscopy images used in this manuscript are openly available in figshare at https://figshare.com/s/55df576890e98cc70105."

Human research participants

Policy information about studies involving human research participants and Sex and Gender in Research.

Reporting on sex and gender	N/A
Population characteristics	N/A
Recruitment	N/A
Ethics oversight	N/A

Note that full information on the approval of the study protocol must also be provided in the manuscript.

Field-specific reporting

Please select the one below that is the best fit for your research. If you are not sure, read the appropriate sections before making your selection.

☒ Life sciences ☐ Behavioural & social sciences ☐ Ecological, evolutionary & environmental sciences

For a reference copy of the document with all sections, see nature.com/documents/nr-reporting-summary-flat.pdf

Life sciences study design

All studies must disclose on these points even when the disclosure is negative.

Sample size	Sample size for was determined according to similar studies in the field. Duncan et al., 2016 Plant Methods (doi: 10.1186/s13007-016-0114-x. eCollection 2016.); Duncan et al., 2022 JXB (doi: 10.1093/jxb/erac521).
Data exclusions	No samples or recordings were excluded.
Replication	All experiments contain at least three biological replicates, with multiple cells in each set. Specific details regarding replication design can be found in figure legends.
Randomization	Seedlings (genotypes or treatments) were always randomly distributed during growth.
Blinding	Image analysis is automated and unbiased. Therefore, no further blinding was required.

Reporting for specific materials, systems and methods

We require information from authors about some types of materials, experimental systems and methods used in many studies. Here, indicate whether each material, system or method listed is relevant to your study. If you are not sure if a list item applies to your research, read the appropriate section before selecting a response.

Materials & experimental systems

n/a	Involved in the study	
☐	☒	Antibodies
☒	☐	Eukaryotic cell lines
☒	☐	Palaeontology and archaeology
☒	☐	Animals and other organisms
☒	☐	Clinical data
☒	☐	Dual use research of concern

Methods

n/a	Involved in the study	
☒	☐	ChIP-seq
☒	☐	Flow cytometry
☒	☐	MRI-based neuroimaging

Antibodies

Antibodies used

A rabbit anti-Histone H4 antibody (AHP418, Bio-rad).
- Secondary antibody (goat Alexa Fluor 488 anti-rabbit; Invitrogen, A-11001).

Validation

These antibodies have been tested before:
J Cell Sci (2002) 115 (23): 4597-4605.;
Plant Cell. 2014 Dec;26(12):4821-33.
